# Improved side-chain torsion potentials for the Amber ff99SB protein force field

**DOI:** 10.1002/prot.22711

**Published:** 2010-03-09

**Authors:** Kresten Lindorff-Larsen, Stefano Piana, Kim Palmo, Paul Maragakis, John L Klepeis, Ron O Dror, David E Shaw

**Affiliations:** 1 D. E. Shaw ResearchNew York, New York 10036; 2 Center for Computational Biology and Bioinformatics, Columbia UniversityNew York, New York 10032

**Keywords:** molecular dynamics simulation, molecular mechanics, NMR, rotamer, side chain, protein dynamics, quantum mechanics, dihedral

## Abstract

Recent advances in hardware and software have enabled increasingly long molecular dynamics (MD) simulations of biomolecules, exposing certain limitations in the accuracy of the force fields used for such simulations and spurring efforts to refine these force fields. Recent modifications to the Amber and CHARMM protein force fields, for example, have improved the backbone torsion potentials, remedying deficiencies in earlier versions. Here, we further advance simulation accuracy by improving the amino acid side-chain torsion potentials of the Amber ff99SB force field. First, we used simulations of model alpha-helical systems to identify the four residue types whose rotamer distribution differed the most from expectations based on Protein Data Bank statistics. Second, we optimized the side-chain torsion potentials of these residues to match new, high-level quantum-mechanical calculations. Finally, we used microsecond-timescale MD simulations in explicit solvent to validate the resulting force field against a large set of experimental NMR measurements that directly probe side-chain conformations. The new force field, which we have termed Amber ff99SB-ILDN, exhibits considerably better agreement with the NMR data. Proteins 2010. © 2010 Wiley-Liss, Inc.

## INTRODUCTION

Molecular dynamics (MD) simulations with atomistic, physics-based force fields offer the potential to gain new insight into the functional mechanisms of biomolecules. The successful application of such simulations may, however, be limited by two major shortcomings. First, computational requirements restrict the amount of time that can be simulated and may thus prevent a simulation from exploring all relevant molecular conformations (the *sampling problem*). Second, inaccuracies in the potential energy function may bias the simulation toward incorrect conformations (the *force field problem*).^1^ Although progress in overcoming the sampling problem could make new and important biological phenomena accessible to computational study for the first time, the success of such efforts is critically dependent on force field quality, because inaccuracies in the physical models used for molecular simulation may produce misleading results even in the absence of any computational limitations. Recent advances in both software and hardware have made possible the simulation of biologically relevant processes with atomistic accuracy on timescales well beyond the microsecond.^1^–^3^ These developments, combined with continuous improvements to enhanced sampling techniques,^4^ have placed ever greater demands on force field accuracy.

As exemplified by recent work on nucleic acids^5^ and proteins,^2^ long MD simulations have been used to highlight deficiencies in existing force fields, leading in turn to the development of new and improved versions.^5^ Although much current development in this area is focused on the inclusion of polarization effects,^6^ polarizable force fields are computationally more expensive than their fixed-charge counterparts, and recent studies suggest that there is still room for improvement of nonpolarizable force fields. Minor yet important modifications to the backbone torsion potentials incorporated in recent versions of the Amber and CHARMM force fields (Amber ff99SB^7^ and the CMAP backbone energy correction to CHARMM22^8^) have led to improvements in accuracy compared with earlier releases, as demonstrated, for example, through the comparison of simulation results to experimental data.^9^

Here, we further refine the Amber ff99SB protein force field by optimizing the χ_1_ torsion potentials for amino acid side chains. Among the torsional degrees of freedom in proteins, the χ_1_ torsions are expected to be second only to the backbone torsions in importance for describing protein energetics, yet the relevant terms in the Amber force field have remained virtually identical for 25 years.^10^–^12^ Because these χ_1_ torsion potentials have not, to our knowledge, been systematically revised since their initial introduction, they seemed a natural target for improvement.

We here focused our efforts on those side chains that displayed the largest deviations from expected behavior and used a three-step procedure to refine the force field. First, we identified putatively problematic residue types by comparing the distribution of χ_1_ dihedrals in simulations of short helical peptides with the corresponding statistics for residues in helices in the Protein Data Bank (PDB).^13^ We found that four residue types (isoleucine, leucine, aspartate, and asparagine) exhibited particularly large deviations from the PDB distribution, suggesting that the ff99SB force field does not model these side chains well. Second, we obtained new χ_1_ torsion potentials for these four residues by fitting force-field parameters to *ab initio* quantum level DF-LMP2^14^,^15^ dihedral scans. Finally, we validated the refined force field using a large set of NMR data containing hundreds of measurements that directly probe the relevant side-chain conformations. We found substantial improvements for all four modified residues, as demonstrated by significantly closer agreement between the rotameric states observed in the simulations and those probed by NMR experiments.

## MATERIALS AND METHODS

### MD simulations of short polyalanine helices

We solvated terminally capped alanine-based helices with the sequence Ace-(Ala)_4_Xaa(Ala)_4_-NMA, where Xaa is any 1 of the 20 naturally occurring amino acids other than Gly, Ala, and Pro, in a cubic box with sides of ∼27Å containing ∼600 water molecules. Protonation states were chosen to correspond to neutral pH. Because the goal was to compare the rotamer distributions observed in MD simulations of these peptides to the distributions observed in helices, we applied a weak restraint to both the φ and ϕ torsion angles to ensure that the peptides stayed helical. These restraints were of the form:

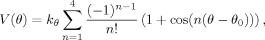

with reference values (θ_0_) of 122° and 133° for φ and ϕ, respectively, and a force constant (*k*_θ_) of 1 kcal mol^−1^. We note that although the reference values do not correspond to the helical region of the Ramachandran map, this cosine series acts as a restraint that ensures that the peptide remains in a helical conformation throughout the entire simulation without noticeably influencing the side-chain motion.

Each system was equilibrated at 300 K and 1 atm with 2.4 ns of MD simulation in the NPT ensemble. Then, MD simulations were carried out in the NVT ensemble for 720 ns using the Nosé-Hoover thermostat with a relaxation time of 1 ps. All simulations were performed using the Desmond MD program^16^ version 2.1.1.0 and either the Amber ff99SB^7^ or the modified Amber ff99SB force field described herein, which we have termed ff99SB-ILDN. All bonds involving hydrogen atoms were constrained with the SHAKE algorithm.^17^ A cutoff of 10 Å was used for the Lennard-Jones interaction and the short-range electrostatic interactions. The smooth particle mesh Ewald method^18^ with a 32 × 32 × 32 grid and a fourth-order interpolation scheme was used to compute the long-range electrostatic interactions. The pairlists were updated every 10 fs with a cutoff of 10.5 Å. We used a multistep RESPA scheme^19^ for the integration of the equations of motion with timesteps of 2.0, 2.0, and 6.0 fs for the bonded, short-range nonbonded, and long-range nonbonded interactions, respectively. To check for potential biases introduced by long-range interactions between peptides in periodic images, we repeated these simulations for four of the amino acids (Xaa: Ile, Leu, Asp, and Asn) using a larger box with side length 37 Å. We found that the results of these control simulations were within error of those using the smaller box sizes.

### MD simulations of small globular proteins

MD simulations of hen egg white lysozyme (HEWL), bovine pancreatic trypsin inhibitor (BPTI), ubiquitin (Ubq), and the B3 domain of Protein G (GB3) were performed using Desmond version 2.1.0.1 and the Amber ff99SB or the modified Amber ff99SB-ILDN force fields. The TIP3P water model^20^ was used for simulations of HEWL, Ubq, and GB3, and the TIP4P-Ew water model^21^ was used for simulations of BPTI. Simulation parameters were the same as in the simulations of small helical peptides, apart from the fact that a 64^3^ PME grid was used for HEWL and a 48^3^ grid was used for BPTI, Ubq, and GB3. Simulations of HEWL, BPTI, Ubq, and GB3 were initiated from PDB^22^ entries 6LYT, 5PTI, 1UBQ, and 1P7E solvated in cubic water boxes containing 10,594, 4215, 6080, and 5156 water molecules, respectively. The net charge of the proteins was neutralized with sodium or chloride ions. Each system was initially subject to energy minimization, followed by 1.2 ns of MD simulation in the NPT ensemble during which the temperature was increased linearly from 10 to 300 K, and position restraints on the backbone atoms were annealed from 1.0 to 0.0 kcal mol^−1^ Å^−1^. After this initial relaxation, each system was simulated for 6 ns in the NPT ensemble. The frame of this simulation with the volume closest to the average volume was selected as the starting conformation for a production run of 1.2 μs in the NVT ensemble. The trajectories obtained from the NVT runs were used for subsequent data analysis.

### Calculation of NMR properties

For all four protein systems, experimentally measured ^3^*J* coupling constants for H^α^–C^α^–C^β^–H^β1,2^ dihedrals are available^23^–^27^ (and Bax, personal communication). The experimental values were compared to those calculated using a Karplus relationship^28^ from the torsion angles observed in the MD simulations. For BPTI, HEWL, and Ubq, stereo-specific assignments allow us to distinguish between couplings for H^β1^ and H^β2^. In GB3, where stereospecific assignments are not available, we used the independently measured C^β^–H^β1,2^ residual dipolar couplings (RDCs) to determine the most likely assignment, as has been suggested previously.^26^ In addition to calculating H^α^–C^α^–C^β^–H^β1,2^ couplings for all four proteins, we also calculated N–C^α^–C^β^–C^γ^ and C′–C^α^–C^β^–C^γ^ couplings in GB3 and Ubq^29^,^30^ and C′–C^α^–C^β^–H^β1,2^ couplings in HEWL.^24^ For the N–C^α^–C^β^–C^γ^ and C′–C^α^–C^β^–C^γ^ couplings in Ile, Val, and Thr, we used Karplus parameters from Chou *et al*.^30^; for all other couplings, we used amino acid–specific parameters from Pérez *et al*.^31^

Side-chain RDCs for GB3 and HEWL were calculated as ensemble averages, as described earlier.^32^ For HEWL, the alignment tensor was first determined using a set of backbone HN RDCs, and the resulting alignment tensor was then used to calculate RDCs for Asn side-chain amides.^33^ As the experiment reports only the sum of the two RDCs for the N^δ^–H^δ1,2^ bonds, we calculated the same sum from the simulations. In GB3, the same procedure was used to determine the alignment tensor from a set of backbone couplings, resulting in a calculation of C^β^–H^β1,2^ couplings.^26^ In total, 390 scalar couplings and 50 RDCs were calculated from the MD simulations and compared to experimental values. The complete dataset, together with the values calculated using ff99SB and ff99SB-ILDN, is available in the Supporting Information for this article.

### *Ab initio* calculations

Quantum mechanical (QM) calculations were performed at the MP2 level of theory, using local and density-fitting approximations,^14^ with an augmented triple-zeta basis set (aug-cc-pVTZ) via the MOLPRO program.^15^ Full scans of the potential energy surface (PES) around the χ_1_ bond were performed for Ace-Xaa-NMA peptides, where Xaa was either Ile, Leu, Asp, or Asn. For each point on the PES, the geometry of the system was fully relaxed with the χ_1_, χ_2_, φ, and ϕ angles constrained. In the Ile and Leu calculations, χ_1_ was varied between −180° and 180° in 15° increments, and for each value of χ_1_, χ_2_ values of −60°, 60°, and 180° were considered. A total of 72 points were optimized for each of these two residues. For Asp and Asn, both χ_1_ and χ_2_ were varied between −180° and 180° in 30° increments. A total of 72 and 144 points were optimized for Asp and Asn, respectively (note that the calculation of the Asp χ_1_/χ_2_ torsion map required half the number of points because of the symmetry of the χ_2_ torsion in Asp). In all calculations, φ and ϕ were kept fixed to the values of −135° and 135°, respectively, corresponding to the extended conformation.

### Parameter fitting

A fit to the potential energy scans was performed by calculating the difference between the molecular mechanics energies and the *ab initio* energies for each point on the PES. The energy terms of the χ_1_ torsion in Ile and Leu and the χ_1_ and χ_2_ torsions in Asp and Asn were then optimized to minimize the following function:

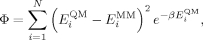

where *E*^MM^ and *E*^QM^ are the force-field and QM energies, respectively, and *N* is the number of conformations optimized at the QM level. The differences between *E*^MM^ and *E*^QM^ are weighted by a Boltzmann factor 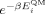. We set the inverse temperature, β = 1/*kT*, to 1.0 mol kcal^−1^ so as to assign to each point a weight that is intermediate between a fit to the energies (β = 0.0 mol kcal^−1^, i.e., uniform weights) and a fit to the Boltzmann populations at room temperature (β ∼1.7 mol kcal^−1^). Our choice of β, corresponding to a temperature of 500 K (β = 1/*kT*), ensures a high level of accuracy at the minima of the energy profile without giving rise to large errors in the barrier regions. The force-field energy, *E*^MM^, is given by the Amber ff99SB energy, *E*^A99SB^, plus a new torsion term, that replaces the existing Amber ff99SB torsion, *V*^A99SB^(θ):


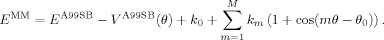


In this equation, *k*_0_ is a constant, the *k*_m_s are the parameters of the fit and represent the force constants for the *M* terms in the cosine expansion, and θ_0_ was fixed to 0.0°, consistent with the Amber force-field convention. Formulated in this way, the resulting parameters define a new torsion potential that is meant to replace the existing torsion term, *V*^A99SB^(θ), in ff99SB. The number of terms, *M*, used in the cosine expansion was two for Ile, three for Leu, and six for both χ_1_ and χ_2_ in Asp and Asn. Allowing for a larger number of parameters in the Ile and Leu torsions did not result in any substantial improvement of the least-squares fit.

## RESULTS

### Comparison of rotamer distributions from MD simulations with the PDB statistics

**Figure 1:**
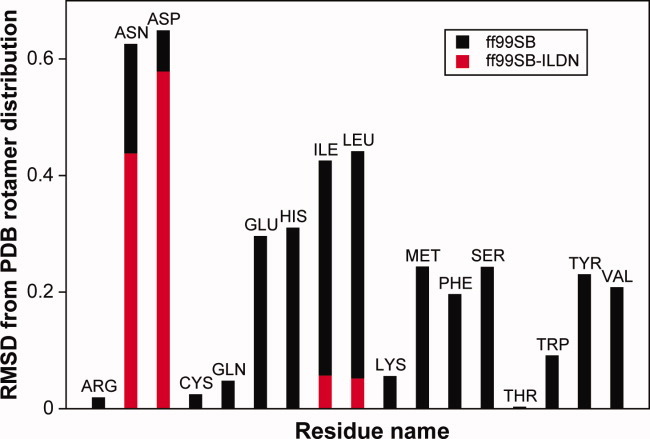
Simulation of small alpha-helical peptides with the Amber ff99SB and ff99SB-ILDN force fields. The plot shows the RMSD between the calculated rotamer distributions for each residue type and the distribution observed for the same residue in helices in the PDB. The values of the χ_1_ dihedral observed in the simulations were partitioned into “plus,” “minus,” or “trans” rotamers as described previously,^13^ and the RMSD was calculated over this three-state classification. The black bars show the results obtained using the ff99SB force field, and the red bars show the results for Ile, Leu, Asp, and Asn using the new side-chain torsion parameters (ff99SB-ILDN) described in this article.

Our approach to the refinement of the Amber ff99SB force field focused on improving the description of the side-chain χ_1_ torsion angle. Previous studies have shown that the distribution of structures in the PDB may be a good approximation for the distributions expected to be observed in an MD simulation.^8^,^34^–^36^ However, such agreements are not expected to be sufficiently quantitative to parameterize force fields, and we instead use comparisons between MD and PDB statistics solely to identify residues whose side chains may be inaccurately described by the force field. More specifically, we performed a series of MD simulations of short helical peptides with the sequences (Ala)_4_Xaa(Ala)_4_, where Xaa is any 1 of the 20 amino acids apart from Gly, Ala, and Pro. From these simulations, we calculated the relative populations of the plus (p), minus (m) and trans (t) χ_1_ rotamers for each residue and compared them to the relative populations observed for the same residue in helices in the PDB^13^ (Fig. 1; see also Supporting Information). This comparison shows clearly that the χ_1_ distributions for four residues (Ile, Leu, Asp, and Asn) differ significantly from those found in the PDB. We find that this result is robust with respect to the similarity metric used to compare the distributions and the length of the simulations. On the basis of these observations, we selected these four residues for refinement of the χ_1_ torsion parameters, as described below. We note here that subsequent comparisons between simulations of proteins and NMR measurements, as described further below, found the same four residues to deviate the most.

### Fitting of torsion potentials to the QM-calculated energies

**Figure 2:**
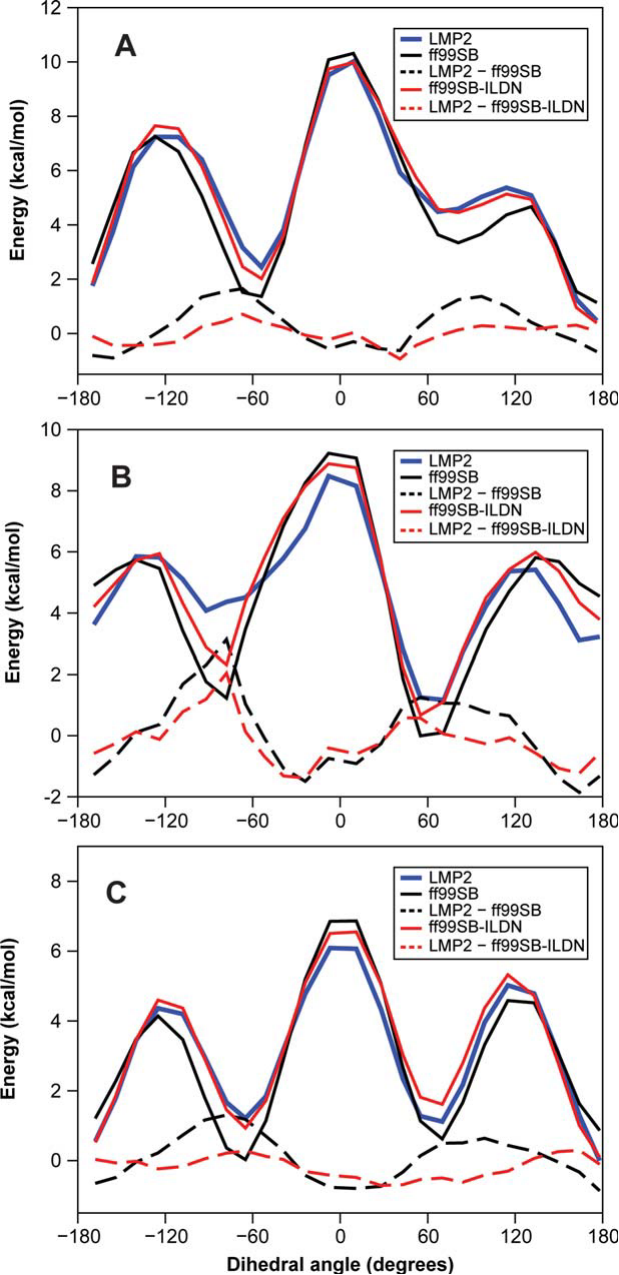
Torsion energy profiles for rotation around the χ_1_ angle in isoleucine. Energy profiles were calculated for three different values of the χ_2_ angle, namely (**A**) −60°, (**B**) 60°, and (**C**) 178°. The dihedral angle shown here is defined as N–C^α^–C^β^–C^γ2^. *Ab initio* energies calculated at the LMP2 level are reported in solid blue lines, whereas Amber ff99SB force field energies are reported in solid black lines. A modified torsion term for the χ_1_ angle (see Table I) was introduced to maximize the agreement between the *ab initio* and the force-field energies. The resulting Amber ff99SB-ILDN energies are reported in solid red lines. The dashed lines show the differences between the QM and molecular mechanics energies (with ff99SB in black dashed lines and ff99SB-ILDN in red dashed lines).

Quantum level *ab initio* calculations at the DF-LMP2 level of theory were used to calculate torsion energy profiles for the four amino acids (Fig. 2 and Supporting Information). As Asp and Asn display more complicated rotameric preferences for χ_2_ than Ile and Leu, and because χ_1_ and χ_2_ torsions appeared to be more strongly coupled in Asp and Asn than in Ile and Leu, we calculated the full two-dimensional χ_1_/χ_2_ energy profile for Asp and Asn and fitted the resulting QM data to new torsion profiles for both χ_1_ and χ_2_. For Ile and Leu, we calculated QM torsion scans for χ_1_ at three values of χ_2_. Although the underlying physical reason for the discrepancies between the QM and force-field energies is not clear, we decided to follow the approach used to modify the ff99SB backbone potential by modifying the torsion energy terms in the force field. The modification of bonded terms such as those for torsion angles will only directly influence a small number of atoms and thereby reduces the possibility of introducing unwanted side effects (when compared with, for example, the modification of nonbonded terms).

In previous studies, force-field torsion parameters have been optimized against quantum chemical calculations using a range of target functions.^7^,^8^,^37^ The choice of target function may affect the resulting force field because it may implicitly weigh different regions of the energy surface differently. In our calculations, we found that it was not possible to obtain sub-kcal mol^−1^ accuracy on a fit to the entire potential energy profile by simply introducing an additional torsion term. For this reason, the direct fit to the energy profile, while giving a good fit to the rotational barrier regions, produces unacceptable errors in the relative rotamer populations. On the other hand, a fit to the room temperature populations introduces substantial errors of up to several kcal mol^−1^ in the barrier regions, as these have negligible Boltzmann factors at room temperature. As described in more detail in the Materials and Methods section, we have thus adopted an intermediate approach of least-square fitting to the Boltzmann population at 500 K. We found that this choice ensures, in practice, that the weights of the high-energy regions, although smaller, are not completely negligible. It also strikes a good balance between the need to obtain good equilibrium populations (residual errors in these regions are typically <0.5 kcal mol^−1^) and reasonable torsion barriers (errors in the barriers are typically between 0.5 and 2 kcal mol^−1^) (Fig. 2 and Supporting Information). The χ_1_ torsion corrections can be introduced on several sets of atoms that would all, in the absence of fluctuations of bond lengths and angles, be related by rotational symmetries. As we, however, decided to follow the convention in the Amber force fields to fix the phase shift, θ_0_, to zero, this symmetry is broken in terms of the resulting torsion terms. For each χ_1_ correction we considered, we therefore fitted to, one at a time, both the N–C^α^–C^β^–C^γ^ and the C′–C^α^–C^β^–C^γ^ dihedral angles and chose the one that gave rise to the best fit to the *ab initio* data. This turned out to be N–C^α^–C^β^–C^γ2^ for Ile, N–C^α^–C^β^–C^γ^ for Asp, and C′–C^α^–C^β^–C^γ^ for Leu and Asn. Adding corrections to more than one of the torsion angles that define χ_1_, or, equivalently, allowing the phase to be nonzero, turned out in practice only to lead to a modest improvement in the quality of the fit, and thus the torsion term for only a single angle was modified. The corrections introduced with this procedure are reported in Table I and range from ∼1 kcal mol^−1^ for Leu up to ∼5 kcal mol^−1^ for Asp. We term the force field that results from replacing the original dihedral terms in ff99SB with these optimized parameters “ff99SB-ILDN” (ILDN being the one-letter code for the side chains whose potentials we modify).

**Table I:** List of Modified Parameters for the χ_1_ and χ_2_ Torsion Potentials in Selected Amino Acids of the Amber ff99SB Force Field

Res.	Angle	θ_0_	*k*_1_	*k*_2_	*k*_3_	*k*_4_	*k*_5_	*k*_6_
Ile	N–C^α^–C^β^–C^γ2^	0.0	0.195	−0.846				
Leu	C–C^α^–C^β^–C^γ^	0.0	0.571	−0.358	0.135			
Asp	N–C^α^–C^β^–C^γ^	0.0	−2.635	−1.190	−0.007	0.423	0.232	−0.213
	C^α^–C^β^–C^γ^–O^δ1,2^	0.0	0.0	−0.443	0.0	−0.138	0.0	−0.013
Asn	C–C^α^–C^β^–C^γ^	0.0	0.571	−0.596	0.118	−0.417	0.104	−0.101
	C^α^–C^β^–C^γ^–N^δ^	0.0	−1.046	−0.181	−0.035	0.100	0.130	−0.106

The parameters are in kcal mol^−1^ and correspond to the torsion potentials that are defined in the main text. Note that for the χ_2_ torsion in Asp, the correction is applied to both side-chain oxygen atoms.

### Rotamer distribution in the ff99SB-ILDN force field

As a first test, we repeated the simulations of the short helical peptides using the modified side-chain torsion potentials. For all four residues that we modified, we find that the ff99SB-ILDN force field improves the agreement with the PDB distribution (Fig. 1; see also Supporting Information). This result is encouraging as the information about the PDB distribution was not used in any way to modify the torsion parameters. We observe a substantial improvement for both Leu and Ile and a marginal one for Asp and Asn. The underlying reason for this difference is not clear. Although even a “perfect” force field would not necessarily reproduce exactly the PDB distribution, the deviations observed for Asp and Asn may be caused by errors in the parametrization of the nonbonded interactions. Such errors cannot be completely compensated for or corrected by introducing a modified torsion potential. As described in the following section, however, we observed substantial improvements for all four residue types when ff99SB and ff99SB-ILDN were evaluated using solution-state NMR measurements.

### Validation through comparison to NMR data

Matching the PDB rotamer distribution is not a direct control that can be used to evaluate the quality of a force field. A more important and stringent test is the ability of the force field to reproduce experimental quantities that directly probe the side-chain conformations of proteins in solution. A large amount of such experimental data is available in the form of NMR side-chain ^3^*J* scalar couplings and RDCs. We have performed MD simulations on the microsecond timescale for four globular proteins (BPTI, ubiquitin, GB3, and lysozyme) in which a large amount of high-quality NMR data probing the side-chain conformation is available. Relatively long simulations are required to achieve a strong convergence of the calculated NMR quantities, as rotameric changes occur on a broad range of timescales. Simulations were performed using both the standard ff99SB force field and the modified ff99SB-ILDN force field. For each of the four proteins, we found the native state to be very stable in the simulations with both ff99SB and ff99SB-ILDN as evidenced, for example, by low average backbone root-mean-square deviations (RMSD) from the experimentally determined structures (≤1 Å in the simulations of BPTI, Ubq, and GB3 and <2 Å in the simulations of HEWL).

**Figure 3:**
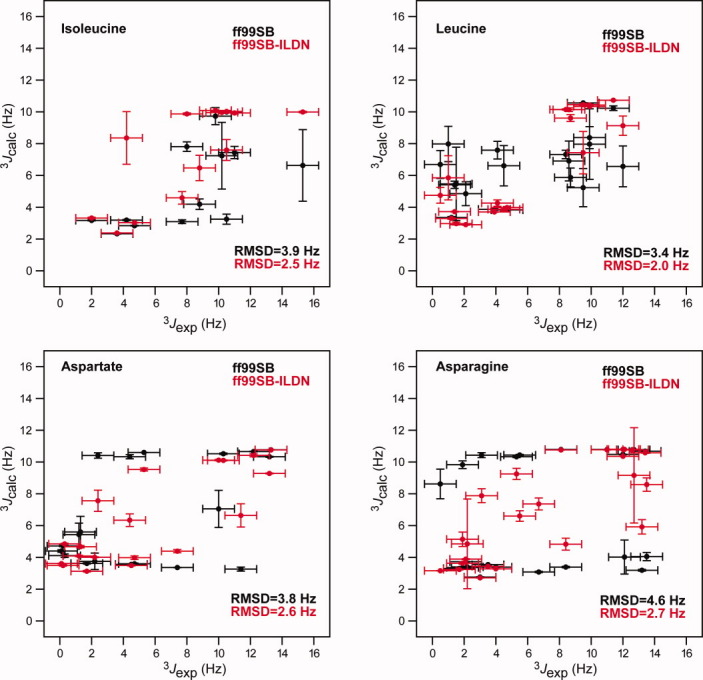
Comparison of experimental NMR ^3^*J* scalar couplings and corresponding values calculated from the MD simulations of HEWL, BPTI, Ubq, and GB3. The plots show H^α^–C^α^–C^β^–H^β1,2^ couplings that directly probe the side-chain χ_1_ angles. Values before (black) and after (red) the side-chain torsion potential refinement are reported for the four residues (Ile, Leu, Asp, and Asn) whose side-chain potentials were modified. Each panel is labeled with the RMSD between the experimental scalar couplings, and the values calculated using the two force fields.

**Figure 4:**
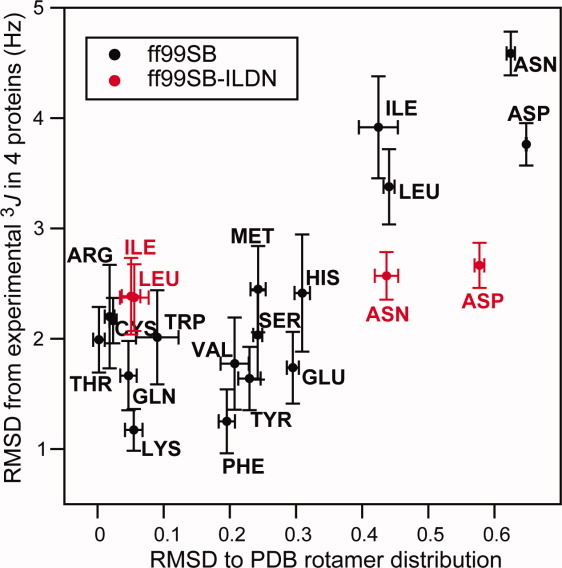
Comparison of different metrics used to evaluate the quality of the side-chain description in force fields. The RMSD between the calculated rotamer distribution and the distribution observed in the PDB is plotted versus the RMSD between the calculated and experimental side-chain NMR ^3^*J* couplings (H^α^–C^α^–C^β^–H^β1,2^). The values calculated after refinement of the side-chain torsion potentials are reported in red.

We calculated a large number of scalar couplings from these simulations and compared them to the experimental values; the results for Ile, Leu, Asp, and Asn are shown in Figure 3. It is clear that many outliers that are present in the simulations with ff99SB are not present in the simulations with ff99SB-ILDN. To quantify the agreement between experiment and simulation, we calculated the RMSD between the experimentally derived and simulation-derived scalar couplings on a per-residue-type basis. The results are shown in Figure 4, where these RMSD values are plotted against the results obtained from the analysis of the helical peptides described above. The results for ff99SB show clearly that the four residues that were selected for force-field refinement based on the comparison between the distribution of rotamers in the PDB and in MD simulations of helical peptides are also the residues that display the largest deviations between the calculated and experimental NMR data. This observation validates our approach of using the deviation from the PDB rotamer distribution in helices as a metric to identify residues that require refinement of their side-chain torsion parameters. In agreement with the visual inspection of Figure 3, the results in Figure 4 show that the description of all four residues that were modified in ff99SB-ILDN improved significantly after the refinement. Notably, for Asp and Asn, the agreement with the NMR data improves much more than the agreement with the PDB rotamer distribution.

**Figure 5:**
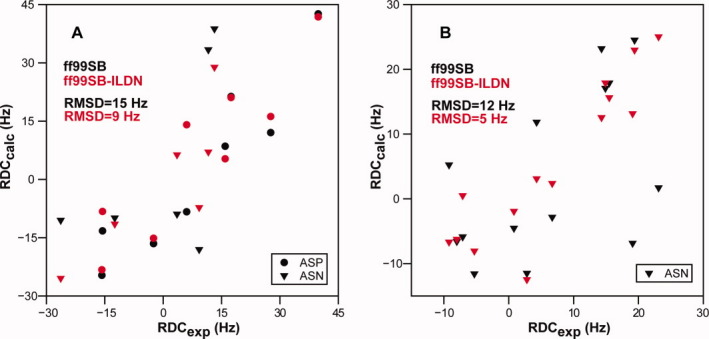
Comparison of experimental residual dipolar couplings and values calculated from the MD simulations of (**A**) GB3 and (**B**) HEWL. In A, we show a comparison between experiment and simulation for C^β^–H^β1,2^ RDCs in GB3 for Asp (circles) and Asn (triangles) residues. In B, we show a comparison between experiment and simulation for N^δ^–H^δ1,2^ RDCs in HEWL for Asn (triangles) residues. The experimental values were reported as the sum of the couplings to the two side-chain amide protons, and so we calculated the same sum from the MD simulations. In both A and B, the results are shown for both simulations with ff99SB (black symbols) and with ff99SB-ILDN (red symbols). Each panel is labeled with the RMSD between the experimental couplings, and the values calculated using the two force fields.

We also compared the simulations to experimentally measured RDCs that act as an alternative probe of the amino acid side-chain orientations. We calculated RDCs for C^β^–H^β1,2^ bonds in GB3 and compared them to the experimental values [Fig. 5(a)]. We observed improvements for both Asp and Asn residues, although these comparisons are complicated by the fact that stereospecific assignments are not available. For HEWL, we compared our simulations to the experimentally measured RDCs for the side-chain amide groups in Asn residues [Fig. 5(b)]. These values depend both on the χ_1_ and χ_2_ torsions and also show significant improvements in the ff99SB-ILDN force field.

## DISCUSSION

We propose a set of improved side-chain torsion parameters for the Amber ff99SB force field. The refinement was limited to the four residues (isoleucine, leucine, aspartate, and asparagine) that appear to be most problematic in ff99SB when comparing the rotamer distribution observed in MD simulation of short helices with the rotamer distribution taken from helices in the PDB. The new parameters were obtained by fitting to new QM data and were validated against a large set of NMR data. The consistent improvements observed for all four of the residues that we modified suggest that our approach is robust and general. Nevertheless, we decided against modifying additional residues, as it would become increasingly difficult to demonstrate significant improvements for those residues. The corrections introduced here for Ile, Leu, Asp, and Asn range between 1 and 5 kcal mol^−1^ and can thus have a noticeable impact on the stability of protein folds and flexible loops, particularly in long MD simulations that exceed the timescales of the rotations of buried or partially buried side chains. Because the new torsion potentials described here represent a clear improvement of those in the existing force field and do not appear to cause undesirable side effects, we recommend the usage of ff99SB-ILDN over ff99SB in MD simulations of proteins.
