# Supplementary material for: Improved side-chain torsion potentials for the Amber ff99SB protein force field
Source: Proteins. 2010 Mar 9;78(8):1950–8. doi: 10.1002/prot.22711 (PMC2970904; doi:10.1002/prot.22711)
Supplement: Supplementary file 1 [file prot0078-1950-SD1.pdf]

Improved side-chain torsion potentials for the Amber ff99SB protein force field

Supporting Information

Kresten Lindorff-Larsen, Stefano Piana, Kim Palmo, Paul Maragakis, John L. Klepeis, Ron O. Dror, and David E. Shaw

Torsion energy profiles for Ile, Leu, Asp and Asn. Four files, one for each amino acid, (`e_torsion_ile.txt`, `e_torsion_leu.txt`, `e_torsion_asp.txt` and `e_torsion_asn.txt`) are provided. In these files, energies (in units of kcal mol^{-1}) are reported as a function of the χ_1 and χ_2 angles. These energies were calculated *ab initio* at the LMP2 level of theory, as well as using the Amber ff99SB force field and the force field containing the improved torsion potentials derived in this study (amber99SB-ILDN). Differences between *ab initio* and force field energies are also reported.

NMR ^{3}J *scalar couplings used for the validation of the amber99SB-ILDN torsion potentials*. Four files (`gb3_3j.txt`, `ubq_3j.txt`, `bpti_3j.txt`, `hewl_3j.txt`) are provided containing the side-chain ^3J scalar couplings used for the validation of the amber99SB-ILDN torsion potentials. Each file contains the experimentally-measured couplings for one of the four proteins used for the validation (GB3, Ubiquitin, BPTI and hen egg white lysozyme) and is reported together with the values calculated in MD simulations using the amber99SB and amber99SB-ILDN force fields.

Rotamer distributions in the PDB and MD simulations of helical peptides. The file `rotamers.txt` contain the rotamer distribution (for the χ_1 dihedral) observed for residues in helices in the PDB as well as in simulations of the poly-alanine-based peptides described in this manuscript.

```
#
# CAPPED ISOLEUCINE PEPTIDE
# Calculated energies (kcal/mol) as a function of chi1 and chi2 angles
#
# NOTE: chi1 is defined as N-CA-CB-CG2
#
# Eqm is the ab initio LMP2 energy
# Emm is the Amber99SB energy
# Emod is the modified Amber99SB energy
#
# chi1   chi2   Eqm     Emm   Eqm-Emm   Emod Eqm-Emod
  67.0  -60.0   4.483   3.636   0.847   4.578  -0.096
  81.0  -60.0   4.588   3.340   1.247   4.454   0.134
  97.0  -60.0   5.033   3.667   1.366   4.743   0.290
 114.0  -60.0   5.370   4.369   1.001   5.134   0.236
 131.0  -60.0   5.086   4.671   0.415   4.940   0.146
 147.0  -60.0   3.394   3.375   0.020   3.145   0.249
 162.0  -60.0   1.262   1.545  -0.283   0.954   0.308
 176.0  -60.0   0.475   1.141  -0.667   0.388   0.087
-170.0  -60.0   1.756   2.563  -0.807   1.854  -0.099
-156.0  -60.0   3.736   4.637  -0.901   4.172  -0.435
-142.0  -60.0   6.137   6.656  -0.519   6.576  -0.439
-127.0  -60.0   7.242   7.258  -0.016   7.652  -0.410
-111.0  -60.0   7.236   6.703   0.533   7.541  -0.305
 -95.0  -60.0   6.414   5.071   1.343   6.166   0.248
 -80.0  -60.0   4.660   3.121   1.540   4.228   0.432
 -67.0  -60.0   3.168   1.517   1.652   2.459   0.709
 -54.0  -60.0   2.443   1.363   1.080   2.018   0.426
 -39.0  -60.0   3.818   3.337   0.480   3.592   0.226
 -24.0  -60.0   6.750   6.918  -0.168   6.808  -0.059
  -8.0  -60.0   9.516  10.083  -0.567   9.742  -0.226
   9.0  -60.0  10.016  10.317  -0.301   9.984   0.033
  26.0  -60.0   8.056   8.608  -0.553   8.541  -0.486
  41.0  -60.0   5.922   6.550  -0.628   6.858  -0.936
  52.0  -60.0   5.314   5.149   0.165   5.752  -0.438
  71.0   60.0   1.170   0.106   1.065   1.114   0.056
  84.0   60.0   2.726   1.672   1.054   2.798  -0.073
 100.0   60.0   4.233   3.463   0.770   4.503  -0.270
 116.0   60.0   5.364   4.714   0.650   5.428  -0.064
 134.0   60.0   5.415   5.812  -0.397   5.985  -0.570
 150.0   60.0   4.301   5.682  -1.381   5.369  -1.067
 164.0   60.0   3.110   4.966  -1.856   4.339  -1.230
 178.0   60.0   3.223   4.547  -1.325   3.787  -0.564
-169.0   60.0   3.621   4.900  -1.279   4.203  -0.581
-154.0   60.0   4.744   5.412  -0.668   4.995  -0.251
-140.0   60.0   5.851   5.740   0.111   5.722   0.129
-124.0   60.0   5.823   5.458   0.365   5.944  -0.121
-108.0   60.0   5.102   3.416   1.686   4.318   0.783
 -92.0   60.0   4.077   1.766   2.311   2.882   1.195
 -78.0   60.0   4.365   1.228   3.137   2.320   2.045
 -65.0   60.0   4.508   3.469   1.039   4.373   0.135
```

-52.0	60.0	5.130	5.211	-0.081	5.814	-0.684
-39.0	60.0	5.786	6.846	-1.060	7.100	-1.315
-24.0	60.0	6.764	8.259	-1.495	8.150	-1.385
-8.0	60.0	8.482	9.226	-0.744	8.885	-0.403
11.0	60.0	8.157	9.071	-0.913	8.756	-0.599
28.0	60.0	5.435	5.695	-0.260	5.673	-0.238
43.0	60.0	2.808	1.859	0.949	2.221	0.587
55.0	60.0	1.252	-0.005	1.257	0.674	0.577
70.0	178.0	1.117	0.619	0.498	1.612	-0.496
84.0	178.0	2.167	1.660	0.507	2.786	-0.619
99.0	178.0	3.957	3.317	0.640	4.370	-0.413
115.0	178.0	5.021	4.584	0.437	5.324	-0.303
133.0	178.0	4.786	4.519	0.267	4.724	0.063
149.0	178.0	3.110	3.142	-0.032	2.856	0.253
164.0	178.0	1.302	1.631	-0.329	1.005	0.297
178.0	178.0	0.000	0.864	-0.864	0.104	-0.104
-168.0	178.0	0.557	1.208	-0.651	0.523	0.034
-154.0	178.0	1.791	2.274	-0.483	1.856	-0.065
-140.0	178.0	3.446	3.483	-0.037	3.465	-0.019
-125.0	178.0	4.362	4.141	0.221	4.597	-0.235
-108.0	178.0	4.198	3.466	0.732	4.368	-0.170
-93.0	178.0	2.970	1.808	1.162	2.918	0.052
-78.0	178.0	1.669	0.363	1.306	1.455	0.214
-65.0	178.0	1.211	0.028	1.183	0.932	0.278
-51.0	178.0	1.839	1.139	0.700	1.716	0.123
-39.0	178.0	3.179	2.894	0.285	3.149	0.031
-24.0	178.0	4.776	5.197	-0.421	5.088	-0.311
-7.0	178.0	6.089	6.854	-0.765	6.506	-0.417
11.0	178.0	6.068	6.864	-0.796	6.550	-0.482
28.0	178.0	4.331	5.066	-0.736	5.044	-0.713
43.0	178.0	2.342	2.669	-0.327	3.032	-0.689
55.0	178.0	1.275	1.135	0.140	1.815	-0.540

```
#
# CAPPED LEUCINE PEPTIDE
# Calculated energies (kcal/mol) as a function of chi1 and chi2 angles
# NOTE: chi1 is defined as C-CA-CB-CG
# Eqm is the ab initio LMP2 energy
# Emm is the Amber99SB energy
# Emod is the modified Amber99SB energy
#
# chi1   chi2   Eqm     Emm   Eqm-Emm   Emod Eqm-Emod
   76.0  -60.0  3.004   3.151  -0.147   3.459  -0.455
   91.0  -60.0  3.320   2.848   0.471   3.148   0.172
  104.0  -60.0  3.687   2.884   0.804   3.096   0.591
  118.0  -60.0  3.770   3.326   0.444   3.336   0.433
  133.0  -60.0  3.214   3.221  -0.007   2.905   0.309
  150.0  -60.0  1.801   2.137  -0.337   1.408   0.393
  165.0  -60.0  0.226   0.855  -0.630  -0.158   0.384
  180.0  -60.0  0.000   1.050  -1.050  -0.070   0.070
 -165.0  -60.0  1.413   3.087  -1.673   2.074  -0.660
 -151.0  -60.0  3.603   5.964  -2.360   5.212  -1.608
 -135.0  -60.0  5.751   8.537  -2.786   8.173  -2.422
 -121.0  -60.0  6.765   9.488  -2.723   9.441  -2.676
 -106.0  -60.0  6.937   8.779  -1.842   8.970  -2.033
  -91.0  -60.0  5.683   7.190  -1.507   7.490  -1.806
  -77.0  -60.0  4.496   5.140  -0.643   5.450  -0.953
  -63.0  -60.0  2.889   3.064  -0.175   3.346  -0.457
  -51.0  -60.0  2.456   1.946   0.510   2.205   0.251
  -40.0  -60.0  3.508   3.048   0.460   3.300   0.208
  -28.0  -60.0  6.037   5.966   0.071   6.229  -0.191
  -11.0  -60.0  8.316   8.617  -0.301   8.903  -0.587
   10.0  -60.0  8.361   8.628  -0.267   8.915  -0.554
   29.0  -60.0  6.133   6.874  -0.742   7.136  -1.003
   45.0  -60.0  3.845   5.063  -1.218   5.316  -1.471
   59.0  -60.0  3.489   3.256   0.232   3.529  -0.040
   75.0   60.0  2.801   2.509   0.292   2.816  -0.015
   90.0   60.0  3.861   2.817   1.044   3.119   0.742
  104.0   60.0  4.130   3.206   0.924   3.418   0.711
  119.0   60.0  4.200   3.604   0.596   3.595   0.605
  136.0   60.0  3.769   4.345  -0.576   3.956  -0.187
  152.0   60.0  2.940   4.236  -1.296   3.462  -0.522
  166.0   60.0  2.369   4.034  -1.665   3.007  -0.638
  180.0   60.0  2.798   4.425  -1.627   3.305  -0.507
 -166.0   60.0  4.119   5.973  -1.853   4.946  -0.827
 -152.0   60.0  5.607   7.794  -2.188   7.020  -1.413
 -137.0   60.0  7.209   9.083  -1.874   8.669  -1.461
 -121.0   60.0  7.578   9.314  -1.736   9.267  -1.689
 -107.0   60.0  7.130   8.153  -1.023   8.332  -1.202
  -94.0   60.0  6.487   6.495  -0.008   6.783  -0.296
  -82.0   60.0  6.037   5.619   0.418   5.933   0.104
  -69.0   60.0  5.650   5.279   0.371   5.575   0.076
  -53.0   60.0  5.768   5.672   0.096   5.933  -0.165
  -37.0   60.0  6.026   5.957   0.069   6.211  -0.185
  -23.0   60.0  7.252   7.292  -0.040   7.561  -0.310
   -7.0   60.0  9.041   8.787   0.254   9.077  -0.036
   13.0   60.0  8.866   8.577   0.289   8.861   0.005
```

32.0	60.0	6.450	6.279	0.171	6.537	-0.087
46.0	60.0	3.699	3.859	-0.161	4.113	-0.414
58.0	60.0	3.056	2.073	0.983	2.343	0.713
72.0	178.0	0.759	0.670	0.089	0.972	-0.213
87.0	178.0	2.402	2.104	0.297	2.414	-0.012
102.0	178.0	3.674	3.475	0.199	3.707	-0.033
119.0	178.0	4.208	4.120	0.088	4.112	0.096
136.0	178.0	3.858	4.465	-0.607	4.076	-0.218
152.0	178.0	3.060	4.473	-1.413	3.699	-0.639
166.0	178.0	2.728	4.624	-1.896	3.597	-0.870
180.0	178.0	3.352	5.366	-2.014	4.246	-0.894
-167.0	178.0	4.516	6.585	-2.069	5.545	-1.029
-154.0	178.0	5.762	7.735	-1.973	6.917	-1.155
-139.0	178.0	6.700	8.513	-1.814	8.050	-1.350
-122.0	178.0	6.862	8.123	-1.261	8.056	-1.194
-108.0	178.0	5.574	5.941	-0.367	6.108	-0.534
-95.0	178.0	4.700	4.091	0.610	4.373	0.327
-83.0	178.0	4.458	3.790	0.667	4.103	0.354
-70.0	178.0	4.816	4.647	0.169	4.945	-0.129
-54.0	178.0	5.465	5.778	-0.312	6.040	-0.575
-36.0	178.0	6.160	6.749	-0.589	7.004	-0.843
-19.0	178.0	6.940	7.328	-0.388	7.604	-0.664
-2.0	178.0	7.828	7.669	0.159	7.961	-0.133
17.0	178.0	6.600	6.683	-0.083	6.961	-0.361
34.0	178.0	3.966	4.038	-0.071	4.293	-0.327
47.0	178.0	1.518	1.380	0.138	1.635	-0.117
58.0	178.0	0.491	-0.001	0.492	0.269	0.222

```
#
# CAPPED ASPARTATE PEPTIDE
# Calculated energies (kcal/mol) as a function of chi1 and chi2 angles
#
# NOTE: chi1 is defined as N-CA-CB-CG
#
# Eqm is the ab initio LMP2 energy
# Emm is the Amber99SB energy
# Emod is the modified Amber99SB energy
#
# chi1   chi2   Eqm     Emm   Eqm-Emm   Emod Eqm-Emod
-150.0 -120.0   8.534   5.738   2.796   6.807   1.727
-120.0 -120.0  14.990  11.938   3.052  12.484   2.506
 -90.0 -120.0  12.540  12.056   0.485  13.061  -0.521
 -60.0 -120.0   8.339  10.295  -1.956   8.451  -0.112
 -30.0 -120.0   7.710  11.367  -3.657   7.471   0.240
  -0.0 -120.0   9.559  13.003  -3.444   8.792   0.767
  30.0 -120.0  12.704  13.632  -0.929   9.736   2.968
  60.0 -120.0  14.472  13.698   0.775  11.853   2.619
  90.0 -120.0  12.132  12.795  -0.663  13.800  -1.668
 120.0 -120.0  10.540  10.966  -0.426  11.511  -0.971
 150.0 -120.0   5.238   5.076   0.162   6.145  -0.907
 178.0 -120.0   1.769   1.052   0.717   1.667   0.102
-150.0  -30.0   1.230   1.595  -0.365   1.830  -0.600
-120.0  -30.0   7.969   8.218  -0.249   7.930   0.039
 -90.0  -30.0  11.940  11.691   0.249  11.863   0.077
 -60.0  -30.0   9.877  13.356  -3.479  10.678  -0.801
 -30.0  -30.0  11.209  15.879  -4.670  11.149   0.061
   0.0  -30.0  12.290  17.035  -4.744  11.990   0.300
  30.0  -30.0   7.481  12.473  -4.992   7.743  -0.262
  60.0  -30.0   7.997  10.470  -2.473   7.793   0.204
  90.0  -30.0  15.868  15.334   0.534  15.506   0.362
 120.0  -30.0  15.573  17.985  -2.411  17.697  -2.124
 150.0  -30.0   7.671  12.423  -4.752  12.658  -4.987
 178.0  -30.0   3.264   4.804  -1.540   4.586  -1.321
-150.0  178.0   1.187   1.504  -0.318   0.836   0.350
-120.0  178.0  10.348  10.315   0.033   9.124   1.224
 -90.0  178.0  12.686  13.936  -1.250  13.205  -0.519
 -60.0  178.0   9.659  14.455  -4.796  10.874  -1.215
 -30.0  178.0  11.247  16.762  -5.515  11.128   0.119
   0.0  178.0  10.253  16.763  -6.511  10.816  -0.563
  30.0  178.0   7.160  13.036  -5.876   7.402  -0.242
  60.0  178.0  10.678  13.088  -2.410   9.507   1.171
  90.0  178.0  16.914  17.911  -0.997  17.179  -0.265
 120.0  178.0  11.715  18.483  -6.769  17.292  -5.578
 150.0  178.0   6.888  10.371  -3.483   9.703  -2.815
 178.0  178.0   0.799   1.965  -1.166   0.843  -0.044
-150.0    0.0   1.392   1.717  -0.325   1.043   0.348
-120.0    0.0  10.886  10.740   0.146   9.543   1.343
 -90.0    0.0  12.771  14.087  -1.315  13.350  -0.579
 -60.0    0.0   9.651  14.472  -4.821  10.886  -1.234
 -30.0    0.0  11.225  16.756  -5.531  11.117   0.108
   0.0    0.0  10.157  16.714  -6.557  10.760  -0.603
  30.0    0.0   7.284  12.999  -5.716   7.360  -0.077
```

60.0	0.0	11.298	13.656	-2.358	10.070	1.228
90.0	0.0	16.897	17.943	-1.045	17.206	-0.309
120.0	0.0	11.737	18.450	-6.714	17.254	-5.517
150.0	0.0	6.821	10.594	-3.773	9.921	-3.100
178.0	0.0	0.748	2.223	-1.475	1.096	-0.348
-150.0	-150.0	4.831	3.815	1.016	4.051	0.780
-120.0	-150.0	13.845	12.546	1.299	12.258	1.587
-90.0	-150.0	12.888	14.182	-1.294	14.354	-1.467
-60.0	-150.0	9.406	13.250	-3.844	10.573	-1.167
-30.0	-150.0	9.344	14.539	-5.194	9.809	-0.464
0.0	-150.0	8.741	14.004	-5.263	8.959	-0.219
30.0	-150.0	9.541	13.308	-3.767	8.578	0.963
60.0	-150.0	14.552	15.424	-0.872	12.746	1.806
90.0	-150.0	14.048	16.773	-2.725	16.945	-2.897
120.0	-150.0	10.164	14.246	-4.082	13.959	-3.794
150.0	-150.0	5.095	6.169	-1.074	6.405	-1.309
178.0	-150.0	0.000	-0.001	0.001	-0.219	0.219
-150.0	-60.0	4.064	3.259	0.805	4.328	-0.264
-120.0	-60.0	8.694	7.170	1.524	7.715	0.978
-90.0	-60.0	11.422	9.401	2.021	10.407	1.016
-60.0	-60.0	9.088	10.767	-1.679	8.923	0.166
-30.0	-60.0	9.949	13.491	-3.542	9.594	0.355
0.0	-60.0	12.955	15.346	-2.391	11.135	1.820
30.0	-60.0	10.300	12.138	-1.838	8.241	2.059
60.0	-60.0	7.230	9.158	-1.928	7.314	-0.084
90.0	-60.0	13.626	11.995	1.631	13.000	0.626
120.0	-60.0	16.341	14.731	1.610	15.277	1.065
150.0	-60.0	8.934	10.622	-1.689	11.691	-2.757
178.0	-60.0	4.692	5.098	-0.406	5.713	-1.021
-150.0	-90.0	7.845	5.252	2.593	6.404	1.441
-120.0	-90.0	13.024	9.696	3.328	10.325	2.699
-90.0	-90.0	11.905	10.008	1.898	11.096	0.809
-60.0	-90.0	7.830	9.061	-1.230	7.300	0.530
-30.0	-90.0	8.010	11.338	-3.328	7.524	0.485
-0.0	-90.0	11.147	13.904	-2.757	9.776	1.371
30.0	-90.0	13.238	12.853	0.385	9.040	4.199
60.0	-90.0	11.122	10.633	0.489	8.872	2.249
90.0	-90.0	12.265	10.802	1.463	11.890	0.374
120.0	-90.0	13.262	11.444	1.817	12.074	1.188
150.0	-90.0	7.268	7.327	-0.059	8.479	-1.211
178.0	-90.0	4.265	3.583	0.681	4.282	-0.017

```
#
# CAPPED ASPARAGINE PEPTIDE
# Calculated energies (kcal/mol) as a function of chi1 and chi2 angles
#
# NOTE: chi1 is defined as C-CA-CB-CG
#       chi2 is defined as CA-CB-CG-ND
#
# Eqm is the ab initio LMP2 energy
# Emm is the Amber99SB energy
# Emod is the modified Amber99SB energy
#
```

# chi1	chi2	Eqm	Emm	Eqm-Emm	Emod	Eqm-Emod
85.0	-120.0	4.184	5.993	-1.809	4.361	-0.177
117.0	-120.0	8.839	11.583	-2.744	9.774	-0.936
151.0	-120.0	7.547	11.215	-3.668	8.786	-1.239
178.0	-120.0	4.440	8.660	-4.220	4.787	-0.347
-155.0	-120.0	5.090	9.592	-4.502	6.904	-1.814
-124.0	-120.0	6.658	11.317	-4.658	9.550	-2.892
-95.0	-120.0	7.039	10.076	-3.037	8.318	-1.279
-66.0	-120.0	6.330	9.263	-2.932	7.857	-1.526
-34.0	-120.0	5.552	9.028	-3.475	7.566	-2.013
-1.0	-120.0	5.637	8.367	-2.730	6.072	-0.435
31.0	-120.0	2.960	4.247	-1.287	2.713	0.247
55.0	-120.0	0.397	1.757	-1.360	0.438	-0.041
87.0	-30.0	5.509	11.512	-6.003	8.371	-2.862
119.0	-30.0	9.096	14.382	-5.286	11.102	-2.006
150.0	-30.0	4.034	10.434	-6.400	6.582	-2.547
179.0	-30.0	0.000	5.437	-5.437	0.073	-0.073
-154.0	-30.0	3.653	8.744	-5.091	4.640	-0.987
-127.0	-30.0	9.532	15.869	-6.337	12.629	-3.096
-93.0	-30.0	7.606	15.235	-7.628	12.016	-4.410
-64.0	-30.0	6.017	10.832	-4.815	7.962	-1.945
-37.0	-30.0	5.146	8.064	-2.918	5.178	-0.032
-7.0	-30.0	7.827	9.647	-1.821	5.929	1.898
27.0	-30.0	7.399	10.088	-2.689	6.956	0.443
58.0	-30.0	5.659	9.353	-3.693	6.532	-0.873
83.0	178.0	0.825	0.913	-0.088	0.317	0.508
115.0	178.0	5.674	7.087	-1.414	6.276	-0.603
151.0	178.0	6.851	10.052	-3.201	8.633	-1.781
-179.0	178.0	6.185	10.523	-4.338	7.651	-1.466
-152.0	178.0	8.556	12.737	-4.181	11.255	-2.699
-123.0	178.0	10.551	13.625	-3.074	12.862	-2.312
-92.0	178.0	6.874	9.863	-2.989	9.150	-2.276
-68.0	178.0	6.480	7.950	-1.470	7.532	-1.052
-41.0	178.0	10.568	12.465	-1.897	12.128	-1.560
-4.0	178.0	9.355	13.277	-3.922	12.010	-2.655
32.0	178.0	5.150	7.265	-2.114	6.765	-1.615
59.0	178.0	0.611	1.320	-0.709	0.985	-0.374
88.0	0.0	6.647	13.077	-6.429	9.309	-2.662
118.0	0.0	5.686	13.048	-7.361	9.149	-3.463
151.0	0.0	1.152	5.756	-4.604	1.231	-0.079
180.0	0.0	0.930	6.559	-5.629	0.579	0.350
-155.0	0.0	7.349	14.737	-7.388	9.953	-2.605
-124.0	0.0	10.125	20.363	-10.238	16.501	-6.376

-91.0	0.0	9.346	16.818	-7.472	13.012	-3.666
-65.0	0.0	2.554	7.207	-4.654	3.715	-1.162
-40.0	0.0	5.769	8.802	-3.033	5.349	0.420
-9.0	0.0	9.715	13.842	-4.126	9.549	0.166
31.0	0.0	8.815	14.864	-6.049	11.234	-2.419
59.0	0.0	5.526	12.540	-7.014	9.100	-3.574
82.0	-150.0	2.362	4.019	-1.656	3.326	-0.964
117.0	-150.0	7.722	10.415	-2.694	9.506	-1.785
151.0	-150.0	7.925	11.469	-3.544	9.940	-2.015
179.0	-150.0	5.890	10.337	-4.447	7.356	-1.466
-154.0	-150.0	7.178	11.630	-4.451	9.909	-2.731
-123.0	-150.0	8.594	12.103	-3.509	11.232	-2.637
-94.0	-150.0	6.912	9.867	-2.954	9.020	-2.108
-69.0	-150.0	7.821	10.062	-2.241	9.525	-1.704
-37.0	-150.0	8.108	11.703	-3.595	11.201	-3.093
-2.0	-150.0	6.876	10.477	-3.601	9.085	-2.209
32.0	-150.0	3.581	5.004	-1.423	4.395	-0.815
57.0	-150.0	0.038	-0.002	0.040	-0.432	0.470
85.0	120.0	1.753	2.295	-0.542	0.664	1.090
113.0	120.0	3.616	5.519	-1.903	3.689	-0.073
148.0	120.0	3.669	7.083	-3.414	4.827	-1.158
179.0	120.0	4.323	8.652	-4.329	4.771	-0.448
-151.0	120.0	6.230	11.625	-5.396	9.196	-2.967
-120.0	120.0	7.552	12.346	-4.795	10.557	-3.005
-90.0	120.0	5.066	8.806	-3.741	7.109	-2.043
-64.0	120.0	2.909	5.789	-2.880	4.402	-1.493
-38.0	120.0	7.734	9.411	-1.677	8.025	-0.291
-7.0	120.0	11.033	13.089	-2.056	10.853	0.179
29.0	120.0	6.263	9.433	-3.170	7.844	-1.581
58.0	120.0	2.353	3.738	-1.384	2.401	-0.048
87.0	-60.0	5.159	7.316	-2.158	5.412	-0.254
117.0	-60.0	7.929	11.586	-3.658	9.532	-1.603
150.0	-60.0	6.835	9.895	-3.060	7.280	-0.445
179.0	-60.0	2.465	6.504	-4.039	2.378	0.088
-154.0	-60.0	2.273	6.499	-4.226	3.632	-1.359
-126.0	-60.0	6.357	9.804	-3.447	7.799	-1.442
-92.0	-60.0	6.959	10.841	-3.882	8.873	-1.914
-63.0	-60.0	3.923	7.954	-4.031	6.330	-2.407
-36.0	-60.0	5.770	7.392	-1.621	5.725	0.045
-4.0	-60.0	6.871	7.162	-0.291	4.639	2.231
29.0	-60.0	4.627	4.582	0.046	2.747	1.881
56.0	-60.0	4.028	4.407	-0.379	2.837	1.191
84.0	90.0	2.266	2.327	-0.061	1.139	1.128
113.0	90.0	3.502	4.572	-1.070	3.172	0.330
149.0	90.0	3.475	6.054	-2.579	4.173	-0.697
178.0	90.0	3.353	6.749	-3.396	3.306	0.047
-152.0	90.0	6.289	10.642	-4.353	8.581	-2.291
-119.0	90.0	5.553	10.433	-4.881	9.067	-3.515
-90.0	90.0	3.676	6.040	-2.364	4.773	-1.097
-65.0	90.0	3.935	5.063	-1.128	4.097	-0.162
-36.0	90.0	6.463	8.182	-1.718	7.191	-0.728
-4.0	90.0	10.174	11.333	-1.160	9.487	0.687
29.0	90.0	6.059	8.244	-2.186	7.086	-1.027
56.0	90.0	2.261	3.717	-1.456	2.823	-0.562

85.0	60.0	2.224	3.778	-1.554	1.900	0.324
115.0	60.0	2.062	2.758	-0.696	0.692	1.370
148.0	60.0	3.110	4.532	-1.422	2.031	1.079
179.0	60.0	3.556	8.769	-5.213	4.642	-1.086
-155.0	60.0	6.864	12.332	-5.468	9.398	-2.534
-119.0	60.0	6.875	12.109	-5.234	10.067	-3.192
-91.0	60.0	2.054	4.064	-2.010	2.109	-0.054
-68.0	60.0	3.695	3.945	-0.250	2.273	1.422
-37.0	60.0	8.601	9.628	-1.027	7.980	0.621
-3.0	60.0	8.997	12.061	-3.064	9.530	-0.533
31.0	60.0	7.290	9.225	-1.935	7.445	-0.155
56.0	60.0	2.178	4.667	-2.489	3.097	-0.920
85.0	150.0	0.534	1.681	-1.147	0.949	-0.415
113.0	150.0	3.744	6.122	-2.378	5.192	-1.448
150.0	150.0	5.011	8.322	-3.311	6.853	-1.842
-179.0	150.0	5.377	10.021	-4.645	7.040	-1.664
-152.0	150.0	7.664	12.454	-4.789	10.862	-3.198
-122.0	150.0	9.308	13.709	-4.402	12.832	-3.525
-91.0	150.0	6.715	9.696	-2.981	8.886	-2.171
-66.0	150.0	4.395	6.720	-2.325	6.214	-1.819
-41.0	150.0	9.675	11.271	-1.596	10.825	-1.150
-7.0	150.0	11.198	14.160	-2.962	12.825	-1.627
30.0	150.0	5.620	9.290	-3.670	8.629	-3.009
59.0	150.0	2.110	2.662	-0.552	2.218	-0.108
85.0	-90.0	5.273	6.249	-0.976	5.047	0.226
117.0	-90.0	8.381	10.944	-2.563	9.565	-1.184
149.0	-90.0	6.659	9.665	-3.006	7.784	-1.125
178.0	-90.0	3.322	6.744	-3.422	3.301	0.021
-155.0	-90.0	3.597	7.721	-4.124	5.463	-1.866
-126.0	-90.0	5.832	8.960	-3.127	7.631	-1.799
-94.0	-90.0	6.607	9.173	-2.565	7.856	-1.249
-64.0	-90.0	4.742	6.335	-1.593	5.378	-0.637
-34.0	-90.0	4.395	6.882	-2.487	5.850	-1.455
-3.0	-90.0	6.187	7.100	-0.913	5.245	0.942
30.0	-90.0	3.145	3.744	-0.599	2.613	0.532
55.0	-90.0	1.558	2.194	-0.636	1.306	0.252
86.0	30.0	3.511	8.362	-4.851	5.233	-1.723
117.0	30.0	2.279	6.617	-4.338	3.325	-1.046
150.0	30.0	0.923	4.026	-3.103	0.173	0.750
179.0	30.0	4.144	8.512	-4.368	3.148	0.997
-154.0	30.0	7.651	16.086	-8.435	11.982	-4.331
-124.0	30.0	9.993	18.084	-8.092	14.834	-4.841
-89.0	30.0	3.376	8.739	-5.363	5.571	-2.195
-68.0	30.0	2.493	4.407	-1.915	1.497	0.995
-41.0	30.0	7.133	9.582	-2.449	6.753	0.380
-4.0	30.0	11.096	15.607	-4.511	11.847	-0.751
31.0	30.0	7.934	13.429	-5.495	10.411	-2.477
60.0	30.0	5.002	9.277	-4.275	6.442	-1.440

```
#
# Scalar coupling data for GB3
#
# HA-CA-CB-HB couplings
# Column 1: Residue number
# Column 2: Four atoms that define the dihedral, each labelled by atom name
# Column 3: Experimental scalar coupling
# Column 4: Calculated scalar coupling from ff99SB simulation
# Column 5: Calculated scalar coupling from ff99SB-ILDN simulation
#
3   HA_CA_CB_HB2                     11.81 10.82 10.82
3   HA_CA_CB_HB3                      1.95  3.42  3.43
8   HA_CA_CB_HB2                      6.09  3.26  7.36
8   HA_CA_CB_HB3                      7.07 10.59  6.62
22  HA_CA_CB_HB2                      3.99  4.04  3.25
22  HA_CA_CB_HB3                      2.13  8.91  5.06
30  HA_CA_CB_HB2                     11.33 10.58 10.56
30  HA_CA_CB_HB3                      0.75  2.68  2.67
35  HA_CA_CB_HB2                      7.92  3.76  5.06
35  HA_CA_CB_HB3                      7.15 10.49  9.05
37  HA_CA_CB_HB2                     12.27  8.48 10.40
37  HA_CA_CB_HB3                      1.94  4.73  3.18
40  HA_CA_CB_HB2                      3.64  3.30  4.15
40  HA_CA_CB_HB3                     10.18 10.57  9.16
43  HA_CA_CB_HB2                     11.13 10.59 10.60
43  HA_CA_CB_HB3                      1.78  2.75  2.75
45  HA_CA_CB_HB2                      4.41  3.68  3.66
45  HA_CA_CB_HB3                     11.18 10.80 10.80
46  HA_CA_CB_HB2                      3.41  3.90  3.86
46  HA_CA_CB_HB3                     11.81 10.74 10.70
47  HA_CA_CB_HB2                      3.58  2.96  3.02
47  HA_CA_CB_HB3                     11.10 10.45 10.35
#
#
# N/C'-CA-CB-CG couplings, for residues where CG is a methyl
# Column 1: Residue number
# Column 2: Residue-name and first & last atom for dihedral (e.g. VAL_CCG1
means C'-CA-CG-CG1 in a Valine)
# Column 3: Experimental scalar coupling
# Column 4: Calculated scalar coupling from ff99SB simulation
# Column 5: Calculated scalar coupling from ff99SB-ILDN simulation
#
6   VAL_CCG1            0.77  0.90  0.92
6   VAL_CCG2            3.68  3.74  3.75
6   VAL_NCG1            1.99  1.92  1.93
6   VAL_NCG2            0.53  0.92  0.92
7   ILE_CCG2            0.58  0.96  0.63
7   ILE_NCG2            1.97  1.61  1.97
11  THR_CCG2            2.11  2.65  2.63
11  THR_NCG2            0.90  0.79  0.78
16  THR_CCG2            1.78  1.43  1.41
16  THR_NCG2            0.21  0.29  0.27
17  THR_CCG2            2.80  3.02  3.00
17  THR_NCG2            0.56  0.67  0.67
18  THR_CCG2            1.69  1.67  1.67
18  THR_NCG2            0.00  0.10  0.08
21  VAL_CCG1            2.52  3.47  3.11
```

21	VAL_CCG2	1.09	1.02	1.14
21	VAL_NCG1	0.69	0.84	0.81
21	VAL_NCG2	1.08	0.48	0.68
25	THR_CCG2	2.60	1.41	1.18
25	THR_NCG2	1.75	1.21	1.19
39	VAL_CCG1	0.86	1.63	1.35
39	VAL_CCG2	3.27	2.93	3.28
39	VAL_NCG1	1.86	1.58	1.73
39	VAL_NCG2	0.58	0.85	0.87
42	VAL_CCG1	1.27	2.43	2.75
42	VAL_CCG2	3.04	1.98	1.76
42	VAL_NCG1	1.73	1.15	1.07
42	VAL_NCG2	0.74	0.74	0.67
44	THR_CCG2	2.88	2.62	2.51
44	THR_NCG2	0.47	0.59	0.59
49	THR_CCG2	3.05	3.50	3.48
49	THR_NCG2	0.77	0.68	0.70
51	THR_CCG2	0.52	0.26	0.26
51	THR_NCG2	1.61	1.60	1.60
53	THR_CCG2	0.45	0.46	0.40
53	THR_NCG2	1.57	1.44	1.47
54	VAL_CCG1	0.70	0.80	0.91
54	VAL_CCG2	1.01	1.68	0.89
54	VAL_NCG1	0.83	0.82	0.41
54	VAL_NCG2	1.85	1.67	1.85
55	THR_CCG2	0.00	0.71	0.58
55	THR_NCG2	1.51	1.32	1.40

```
#
# Scalar coupling data for Ubq
#
# HA-CA-CB-HB couplings
# Column 1: Residue number
# Column 2: Four atoms that define the dihedral, each labelled by residue
number and atom name
# Column 3: Experimental scalar coupling
# Column 4: Calculated scalar coupling from ff99SB simulation
# Column 5: Calculated scalar coupling from ff99SB-ILDN simulation
#
1   1_HA_1_CA_1_CB_1_1HB       4.30  4.08  4.19
3   3_HA_3_CA_3_CB_3_HB        4.70  2.84  3.03
4   4_HA_4_CA_4_CB_4_1HB      11.90 10.57 10.54
4   4_HA_4_CA_4_CB_4_2HB       3.60  3.99  4.09
5   5_HA_5_CA_5_CB_5_HB        9.60  9.42  8.94
6   6_HA_6_CA_6_CB_6_1HB       6.20  6.80  7.41
6   6_HA_6_CA_6_CB_6_2HB       7.00  6.62  6.00
8   8_HA_8_CA_8_CB_8_1HB       8.70  5.87  9.35
8   8_HA_8_CA_8_CB_8_2HB       4.10  7.59  4.50
11  11_HA_11_CA_11_CB_11_1HB   5.70  7.15  6.93
11  11_HA_11_CA_11_CB_11_2HB   6.70  5.21  5.50
13  13_HA_13_CA_13_CB_13_HB    7.70  3.09  4.61
15  15_HA_15_CA_15_CB_15_1HB   8.40  7.32 10.18
15  15_HA_15_CA_15_CB_15_2HB   1.40  5.41  3.68
16  16_HA_16_CA_16_CB_16_1HB   5.50  6.46  6.36
16  16_HA_16_CA_16_CB_16_2HB   8.20  7.18  7.28
17  17_HA_17_CA_17_CB_17_HB    2.70  2.87  2.81
18  18_HA_18_CA_18_CB_18_1HB   9.30  6.86  8.83
18  18_HA_18_CA_18_CB_18_2HB   2.50  6.50  4.40
19  19_HA_19_CA_19_CB_19_1HB   6.80  7.07  8.00
19  19_HA_19_CA_19_CB_19_2HB   7.30  6.78  6.71
20  20_HA_20_CA_20_CB_20_1HB   9.40  6.69  5.51
20  20_HA_20_CA_20_CB_20_2HB   3.00  3.15  3.27
23  23_HA_23_CA_23_CB_23_HB   10.20  7.24  9.93
25  25_HA_25_CA_25_CB_25_1HB   8.40  3.39  4.71
25  25_HA_25_CA_25_CB_25_2HB   5.30 10.33  9.39
26  26_HA_26_CA_26_CB_26_HB    9.90 10.04 10.02
27  27_HA_27_CA_27_CB_27_1HB  10.60 10.68 10.64
27  27_HA_27_CA_27_CB_27_2HB   2.40  2.93  2.85
29  29_HA_29_CA_29_CB_29_1HB   9.20 10.24 10.34
29  29_HA_29_CA_29_CB_29_2HB   1.90  3.07  2.96
30  30_HA_30_CA_30_CB_30_HB   10.50  9.99 10.03
31  31_HA_31_CA_31_CB_31_1HB   3.80  3.29  3.22
31  31_HA_31_CA_31_CB_31_2HB  11.30 10.68 10.65
32  32_HA_32_CA_32_CB_32_1HB   7.40  3.36  4.42
32  32_HA_32_CA_32_CB_32_2HB   5.30 10.60  9.51
33  33_HA_33_CA_33_CB_33_1HB   9.00  8.61  8.54
33  33_HA_33_CA_33_CB_33_2HB   4.00  4.83  4.98
34  34_HA_34_CA_34_CB_34_1HB  10.20  9.69 10.02
34  34_HA_34_CA_34_CB_34_2HB   4.40  3.61  3.39
36  36_HA_36_CA_36_CB_36_HB    8.80  4.19  7.02
37  37_HA_37_CA_37_CB_37_1HB   6.50  5.65  6.81
37  37_HA_37_CA_37_CB_37_2HB   7.00  6.85  6.83
38  38_HA_38_CA_38_CB_38_1HB   7.80  9.11  7.68
38  38_HA_38_CA_38_CB_38_2HB   5.40  6.23  6.46
39  39_HA_39_CA_39_CB_39_1HB   4.70  3.60  4.01
```

```
39  39_HA_39_CA_39_CB_39_2HB   4.40 10.33  6.08
40  40_HA_40_CA_40_CB_40_1HB  12.20  9.50  9.17
40  40_HA_40_CA_40_CB_40_2HB   2.00  4.26  4.70
42  42_HA_42_CA_42_CB_42_1HB   8.30  5.57  4.55
42  42_HA_42_CA_42_CB_42_2HB   4.30  7.82  8.95
43  43_HA_43_CA_43_CB_43_1HB   9.90  7.97 10.35
43  43_HA_43_CA_43_CB_43_2HB   1.50  5.47  2.93
44  44_HA_44_CA_44_CB_44_HB    8.00  7.81  9.89
45  45_HA_45_CA_45_CB_45_1HB   3.50  3.46  3.34
45  45_HA_45_CA_45_CB_45_2HB   9.80 10.68 10.75
50  50_HA_50_CA_50_CB_50_1HB  11.40 10.23 10.73
50  50_HA_50_CA_50_CB_50_2HB   3.90  3.87  3.71
51  51_HA_51_CA_51_CB_51_1HB   9.20  9.31  6.61
51  51_HA_51_CA_51_CB_51_2HB   3.20  4.41  7.22
52  52_HA_52_CA_52_CB_52_1HB  10.00  7.05 10.14
52  52_HA_52_CA_52_CB_52_2HB   1.30  5.60  4.69
54  54_HA_54_CA_54_CB_54_1HB  11.30  9.93 10.28
54  54_HA_54_CA_54_CB_54_2HB   1.40  3.69  3.44
55  55_HA_55_CA_55_CB_55_HB    2.40  2.06  2.17
56  56_HA_56_CA_56_CB_56_1HB   9.90  8.38 10.41
56  56_HA_56_CA_56_CB_56_2HB   2.10  4.85  2.90
58  58_HA_58_CA_58_CB_58_1HB  11.40  3.26  6.26
58  58_HA_58_CA_58_CB_58_2HB   2.40 10.41  7.90
59  59_HA_59_CA_59_CB_59_1HB  10.00 10.34 10.52
59  59_HA_59_CA_59_CB_59_2HB   1.80  2.81  2.93
60  60_HA_60_CA_60_CB_60_1HB   6.70  3.08  7.34
60  60_HA_60_CA_60_CB_60_2HB   5.50 10.44  6.62
61  61_HA_61_CA_61_CB_61_HB    9.80  9.73 10.08
62  62_HA_62_CA_62_CB_62_1HB  11.10  9.80  9.48
62  62_HA_62_CA_62_CB_62_2HB   3.10  3.69  3.55
63  63_HA_63_CA_63_CB_63_1HB   3.50  4.33  4.38
63  63_HA_63_CA_63_CB_63_2HB   4.40  4.91  4.50
64  64_HA_64_CA_64_CB_64_1HB   9.90 10.33 10.39
64  64_HA_64_CA_64_CB_64_2HB   1.50  3.43  3.37
65  65_HA_65_CA_65_CB_65_1HB   8.90  7.53  8.16
65  65_HA_65_CA_65_CB_65_2HB   4.20  2.84  2.87
66  66_HA_66_CA_66_CB_66_HB   10.60  9.10  9.03
68  68_HA_68_CA_68_CB_68_1HB  10.10  8.00  8.45
68  68_HA_68_CA_68_CB_68_2HB   2.00  5.28  4.69
69  69_HA_69_CA_69_CB_69_1HB   4.70  3.83  3.96
69  69_HA_69_CA_69_CB_69_2HB   9.50 10.56 10.43
70  70_HA_70_CA_70_CB_70_HB    7.30  4.65  3.23
72  72_HA_72_CA_72_CB_72_1HB   6.40  3.95  3.71
72  72_HA_72_CA_72_CB_72_2HB   6.60  8.44  9.04
73  73_HA_73_CA_73_CB_73_1HB   8.60  6.91 10.08
73  73_HA_73_CA_73_CB_73_2HB   4.50  6.61  3.89
#
#
# N/C'-CA-CB-CG couplings, for residues where CG is a methyl
# Column 1: Residue number
# Column 2: Residue-name and first & last atom for dihedral (e.g. ILE_CCG2
means C'-CA-CG-CG2 in an Isoleucine)
# Column 3: Experimental scalar coupling
# Column 4: Calculated scalar coupling from ff99SB simulation
# Column 5: Calculated scalar coupling from ff99SB-ILDN simulation
#
3   ILE_CCG2            3.68  4.08  4.10
```

```
3   ILE_NCG2            0.39  0.58  0.69
5   VAL_CCG1            0.00  0.61  0.64
5   VAL_CCG2            3.66  3.70  3.47
5   VAL_NCG1            1.83  1.85  1.73
5   VAL_NCG2            0.46  1.21  1.24
7   THR_CCG2            2.67  3.25  3.29
7   THR_NCG2            1.08  0.90  0.89
9   THR_CCG2            3.00  3.39  3.40
9   THR_NCG2            0.81  0.60  0.61
12  THR_CCG2            0.39  0.35  0.35
12  THR_NCG2            1.61  1.43  1.42
13  ILE_CCG2            1.71  3.96  3.28
13  ILE_NCG2            1.44  0.70  1.05
14  THR_CCG2            0.76  0.82  0.55
14  THR_NCG2            1.50  1.28  1.36
17  VAL_CCG1            3.93  4.04  4.02
17  VAL_CCG2            0.96  0.77  0.75
17  VAL_NCG1            0.25  0.68  0.63
17  VAL_NCG2            0.68  0.40  0.46
22  THR_CCG2            3.39  2.99  3.46
22  THR_NCG2            0.75  0.74  0.59
23  ILE_CCG2            0.94  1.17  1.17
23  ILE_NCG2            2.11  1.33  2.08
26  VAL_CCG1            0.82  0.66  0.86
26  VAL_CCG2            4.18  4.03  4.05
26  VAL_NCG1            2.16  2.01  2.05
26  VAL_NCG2            0.62  1.05  0.83
30  ILE_CCG2            0.96  1.20  1.16
30  ILE_NCG2            2.10  2.11  2.12
36  ILE_CCG2            0.71  1.78  0.65
36  ILE_NCG2            2.08  0.76  1.36
44  ILE_CCG2            0.80  1.71  0.85
44  ILE_NCG2            1.62  1.63  2.04
55  THR_CCG2            3.02  3.36  3.39
55  THR_NCG2            0.82  0.72  0.83
61  ILE_CCG2            0.98  1.00  0.90
61  ILE_NCG2            2.15  2.02  2.10
70  VAL_CCG2            2.43  1.57  0.93
70  VAL_NCG2            0.50  0.56  0.44
#
#
# C'-CA-CB-CG couplings, for all residues
# Column 1: Residue number
# Column 2: Four atoms that define the dihedral, each labelled by residue
number and atom name
# Column 3: Experimental scalar coupling
# Column 4: Calculated scalar coupling from ff99SB simulation
# Column 5: Calculated scalar coupling from ff99SB-ILDN simulation
#
2   2_C_2_CA_2_CB_2_CG            2.40  1.79  2.18
3   3_C_3_CA_3_CB_3_CG1           1.00  0.50  0.54
3   3_C_3_CA_3_CB_3_CG2           3.80  3.45  3.48
4   4_C_4_CA_4_CB_4_CG            4.00  3.58  3.58
5   5_C_5_CA_5_CB_5_CG2           3.50  3.29  3.09
6   6_C_6_CA_6_CB_6_CG            2.00  2.08  2.32
7   7_C_7_CA_7_CB_7_CG2           2.50  3.24  3.27
8   8_C_8_CA_8_CB_8_CG            2.60  1.67  3.04
```

9	9_C_9_CA_9_CB_9_CG2	2.80	3.08	3.10
13	13_C_13_CA_13_CB_13_CG1	2.30	0.59	1.20
13	13_C_13_CA_13_CB_13_CG2	1.50	3.35	2.80
14	14_C_14_CA_14_CB_14_CG2	0.70	0.79	0.55
15	15_C_15_CA_15_CB_15_CG	2.20	2.27	3.41
16	16_C_16_CA_16_CB_16_CG	1.80	1.93	1.89
17	17_C_17_CA_17_CB_17_CG1	3.60	3.47	3.45
17	17_C_17_CA_17_CB_17_CG2	0.90	0.54	0.54
19	19_C_19_CA_19_CB_19_CG	2.20	2.31	2.65
21	21_C_21_CA_21_CB_21_CG	5.50	1.30	3.91
22	22_C_22_CA_22_CB_22_CG2	3.10	2.78	3.18
24	24_C_24_CA_24_CB_24_CG	1.50	1.92	0.88
25	25_C_25_CA_25_CB_25_CG	3.40	1.54	2.07
26	26_C_26_CA_26_CB_26_CG1	0.80	0.67	0.79
26	26_C_26_CA_26_CB_26_CG2	4.00	3.53	3.50
29	29_C_29_CA_29_CB_29_CG	4.00	3.35	3.39
31	31_C_31_CA_31_CB_31_CG	0.60	0.72	0.70
32	32_C_32_CA_32_CB_32_CG	3.30	1.24	1.66
33	33_C_33_CA_33_CB_33_CG	2.80	2.74	2.72
34	34_C_34_CA_34_CB_34_CG	2.90	3.13	3.27
38	38_C_38_CA_38_CB_38_CG	2.70	3.04	2.53
39	39_C_39_CA_39_CB_39_CG	1.80	1.29	1.42
40	40_C_40_CA_40_CB_40_CG	3.40	3.14	3.03
41	41_C_41_CA_41_CB_41_CG	3.50	3.62	3.44
42	42_C_42_CA_42_CB_42_CG	2.40	1.53	1.17
43	43_C_43_CA_43_CB_43_CG	3.00	2.47	3.34
44	44_C_44_CA_44_CB_44_CG1	3.10	2.56	3.42
44	44_C_44_CA_44_CB_44_CG2	0.70	1.48	0.75
48	48_C_48_CA_48_CB_48_CG	2.50	2.82	2.69
49	49_C_49_CA_49_CB_49_CG	1.40	1.64	2.12
50	50_C_50_CA_50_CB_50_CG	2.40	3.43	3.65
51	51_C_51_CA_51_CB_51_CG	2.90	3.05	2.00
54	54_C_54_CA_54_CB_54_CG	3.50	3.30	3.45
55	55_C_55_CA_55_CB_55_CG2	2.80	3.18	3.27
56	56_C_56_CA_56_CB_56_CG	2.80	2.62	3.42
58	58_C_58_CA_58_CB_58_CG	5.60	1.21	2.40
59	59_C_59_CA_59_CB_59_CG	3.60	3.26	3.35
60	60_C_60_CA_60_CB_60_CG	2.00	1.49	3.14
61	61_C_61_CA_61_CB_61_CG1	3.30	3.35	3.50
61	61_C_61_CA_61_CB_61_CG2	0.80	0.86	0.77
63	63_C_63_CA_63_CB_63_CG	1.20	0.87	0.88
64	64_C_64_CA_64_CB_64_CG	3.40	3.41	3.43
66	66_C_66_CA_66_CB_66_CG2	0.50	0.38	0.38
67	67_C_67_CA_67_CB_67_CG	3.40	3.37	3.49
68	68_C_68_CA_68_CB_68_CG	0.80	2.95	3.12
69	69_C_69_CA_69_CB_69_CG	0.80	0.84	0.89
72	72_C_72_CA_72_CB_72_CG	1.60	0.93	0.92
73	73_C_73_CA_73_CB_73_CG	2.80	2.07	3.34
74	74_C_74_CA_74_CB_74_CG	2.20	2.62	2.54

```
#
# Scalar coupling data for BPTI
#
# HA-CA-CB-HB couplings
# Column 1: Residue number
# Column 2: Four atoms that define the dihedral, each labelled by residue
number and atom name
# Column 3: Experimental scalar coupling
# Column 4: Calculated scalar coupling from ff99SB simulation
# Column 5: Calculated scalar coupling from ff99SB-ILDN simulation
#
4   4_HA_4_CA_4_CB_4_2HB       4.50  3.69  3.90
4   4_HA_4_CA_4_CB_4_HB3       4.50  4.34  4.11
5   5_HA_5_CA_5_CB_5_HB3       3.00  2.79  2.71
5   5_HA_5_CA_5_CB_5_2HB      12.00 10.08 10.07
11  11_HA_11_CA_11_CB_11_HB    8.20  7.73  8.23
14  14_HA_14_CA_14_CB_14_HB3   3.00  2.16  2.26
14  14_HA_14_CA_14_CB_14_2HB  12.00  9.84  9.94
15  15_HA_15_CA_15_CB_15_HB3   3.50  4.13  4.18
15  15_HA_15_CA_15_CB_15_2HB  11.00  8.94  8.85
18  18_HA_18_CA_18_CB_18_HB   10.50  3.25  7.56
19  19_HA_19_CA_19_CB_19_HB   11.00  7.44  9.91
20  20_HA_20_CA_20_CB_20_HB3   2.50  2.71  2.77
20  20_HA_20_CA_20_CB_20_2HB  12.50 10.56 10.63
22  22_HA_22_CA_22_CB_22_HB3   3.50  2.61  2.61
22  22_HA_22_CA_22_CB_22_2HB   4.40  5.74  5.72
23  23_HA_23_CA_23_CB_23_HB3  12.50 10.82 10.83
23  23_HA_23_CA_23_CB_23_2HB   3.80  3.21  3.23
24  24_HA_24_CA_24_CB_24_HB3  12.00 10.80 10.80
24  24_HA_24_CA_24_CB_24_2HB   3.50  3.54  3.44
30  30_HA_30_CA_30_CB_30_2HB  12.00  9.87  9.82
30  30_HA_30_CA_30_CB_30_HB3   2.50  2.14  2.10
31  31_HA_31_CA_31_CB_31_HB3   3.50  3.41  3.28
31  31_HA_31_CA_31_CB_31_2HB  11.00  9.77 10.07
32  32_HA_32_CA_32_CB_32_HB    2.50  2.55  2.58
33  33_HA_33_CA_33_CB_33_HB3   3.00  2.61  2.62
33  33_HA_33_CA_33_CB_33_2HB   4.50  5.78  5.75
34  34_HA_34_CA_34_CB_34_HB   10.50  8.02  7.98
35  35_HA_35_CA_35_CB_35_HB3  11.50 10.42 10.41
35  35_HA_35_CA_35_CB_35_2HB   6.50  4.40  4.40
38  38_HA_38_CA_38_CB_38_2HB   6.50  3.96  4.04
38  38_HA_38_CA_38_CB_38_HB3   1.50  2.45  2.40
41  41_HA_41_CA_41_CB_41_HB3   2.50  3.15  3.17
41  41_HA_41_CA_41_CB_41_2HB  12.50 10.67 10.66
43  43_HA_43_CA_43_CB_43_2HB   3.00  2.76  2.71
43  43_HA_43_CA_43_CB_43_HB3  12.00 10.48 10.37
44  44_HA_44_CA_44_CB_44_2HB   4.00  3.39  3.28
44  44_HA_44_CA_44_CB_44_HB3  11.00 10.79 10.77
45  45_HA_45_CA_45_CB_45_HB3   4.00  3.93  3.92
45  45_HA_45_CA_45_CB_45_2HB  11.80 10.68 10.68
46  46_HA_46_CA_46_CB_46_HB3   4.00  4.46  4.63
46  46_HA_46_CA_46_CB_46_2HB   9.50  8.07  8.04
47  47_HA_47_CA_47_CB_47_2HB   3.00  3.36  3.54
47  47_HA_47_CA_47_CB_47_HB3   2.80  2.72  2.70
51  51_HA_51_CA_51_CB_51_HB3  11.50  9.94  9.93
51  51_HA_51_CA_51_CB_51_2HB   5.50  3.29  3.32
52  52_HA_52_CA_52_CB_52_2HB   3.00  6.15  6.42
```

52	52_HA_52_CA_52_CB_52_HB3	10.20	7.19	6.91
54	54_HA_54_CA_54_CB_54_HB	10.00	8.38	8.77
55	55_HA_55_CA_55_CB_55_2HB	11.00	9.93	9.91
55	55_HA_55_CA_55_CB_55_HB3	1.50	2.28	2.33

```
#
# Scalar coupling data for HEWL
#
# HA-CA-CB-HB couplings
# Column 1: Residue number
# Column 2: Four atoms that define the dihedral, each labelled by residue
number and atom name
# Column 3: Experimental scalar coupling
# Column 4: Calculated scalar coupling from ff99SB simulation
# Column 5: Calculated scalar coupling from ff99SB-ILDN simulation
#
2   2_HA_2_CA_2_CB_2_HB         9.80  7.51  7.29
3   3_HA_3_CA_3_CB_3_1HB        7.70  8.52  9.90
3   3_HA_3_CA_3_CB_3_2HB        1.00  4.15  2.88
6   6_HA_6_CA_6_CB_6_1HB       12.60 10.09 10.07
6   6_HA_6_CA_6_CB_6_2HB        0.90  2.62  2.68
8   8_HA_8_CA_8_CB_8_1HB        1.20  3.35  3.35
12  12_HA_12_CA_12_CB_12_1HB  12.60 10.43 10.46
12  12_HA_12_CA_12_CB_12_2HB   1.00  2.70  2.73
15  15_HA_15_CA_15_CB_15_1HB   9.00 10.46 10.51
15  15_HA_15_CA_15_CB_15_2HB   0.60  3.05  2.99
17  17_HA_17_CA_17_CB_17_1HB   9.50  5.23  5.77
17  17_HA_17_CA_17_CB_17_2HB   1.00  7.98  7.55
18  18_HA_18_CA_18_CB_18_1HB   1.70  3.62  3.21
18  18_HA_18_CA_18_CB_18_2HB  13.20 10.33  9.72
20  20_HA_20_CA_20_CB_20_1HB   1.40  3.39  3.47
20  20_HA_20_CA_20_CB_20_2HB  12.00 10.76 10.76
23  23_HA_23_CA_23_CB_23_1HB   8.30 10.06  8.33
23  23_HA_23_CA_23_CB_23_2HB   0.20  3.82  5.43
24  24_HA_24_CA_24_CB_24_1HB   1.00  5.21  5.98
27  27_HA_27_CA_27_CB_27_1HB  12.10  4.02 10.82
27  27_HA_27_CA_27_CB_27_2HB   0.50  8.62  3.18
30  30_HA_30_CA_30_CB_30_1HB   0.20  2.44  2.97
30  30_HA_30_CA_30_CB_30_2HB   6.80 10.06 10.07
33  33_HA_33_CA_33_CB_33_1HB   2.10  3.49  3.50
33  33_HA_33_CA_33_CB_33_2HB   7.90 10.73 10.70
34  34_HA_34_CA_34_CB_34_1HB  12.10 10.74  9.12
34  34_HA_34_CA_34_CB_34_2HB   1.60  3.10  4.88
38  38_HA_38_CA_38_CB_38_1HB  11.90 10.58 10.55
39  39_HA_39_CA_39_CB_39_1HB   2.20  3.40  4.23
39  39_HA_39_CA_39_CB_39_2HB  12.70 10.78  9.76
41  41_HA_41_CA_41_CB_41_1HB  10.00  9.06  8.51
41  41_HA_41_CA_41_CB_41_2HB   1.20  4.40  4.30
43  43_HA_43_CA_43_CB_43_HB    3.90  2.56  2.98
47  47_HA_47_CA_47_CB_47_HB    2.40  2.87  3.13
48  48_HA_48_CA_48_CB_48_1HB   0.20  4.72  3.49
48  48_HA_48_CA_48_CB_48_2HB   1.20  5.43  4.09
51  51_HA_51_CA_51_CB_51_HB   13.30  8.99  9.17
52  52_HA_52_CA_52_CB_52_1HB  10.30 10.52 10.08
52  52_HA_52_CA_52_CB_52_2HB   0.30  4.11  4.86
53  53_HA_53_CA_53_CB_53_1HB  12.50 10.66 10.74
53  53_HA_53_CA_53_CB_53_2HB   0.80  2.85  2.96
55  55_HA_55_CA_55_CB_55_HB    3.60  2.33  2.40
59  59_HA_59_CA_59_CB_59_1HB   1.90  3.36  3.61
59  59_HA_59_CA_59_CB_59_2HB   8.10 10.79 10.79
64  64_HA_64_CA_64_CB_64_1HB   0.90  3.55  3.63
64  64_HA_64_CA_64_CB_64_2HB   0.50  2.68  2.62
```

```
65  65_HA_65_CA_65_CB_65_1HB   2.10  3.73  3.87
65  65_HA_65_CA_65_CB_65_2HB  13.40 10.68 10.57
66  66_HA_66_CA_66_CB_66_1HB   0.10  4.40  3.64
66  66_HA_66_CA_66_CB_66_2HB   2.20  3.75  3.99
69  69_HA_69_CA_69_CB_69_HB   11.40  9.06  8.85
74  74_HA_74_CA_74_CB_74_1HB   1.70  3.30  3.22
74  74_HA_74_CA_74_CB_74_2HB  12.60 10.78 10.76
80  80_HA_80_CA_80_CB_80_1HB  13.80 10.10 10.10
80  80_HA_80_CA_80_CB_80_2HB   1.00  2.73  2.73
84  84_HA_84_CA_84_CB_84_1HB  12.00  6.57  8.83
84  84_HA_84_CA_84_CB_84_2HB   0.50  6.69  4.98
87  87_HA_87_CA_87_CB_87_2HB  12.20 10.66 10.36
88  88_HA_88_CA_88_CB_88_HB    2.00  3.17  3.27
89  89_HA_89_CA_89_CB_89_HB   10.80  8.30  8.89
93  93_HA_93_CA_93_CB_93_1HB  13.50  4.05  8.30
93  93_HA_93_CA_93_CB_93_2HB   1.90  9.83  5.42
94  94_HA_94_CA_94_CB_94_1HB   2.10  2.64  2.70
94  94_HA_94_CA_94_CB_94_2HB  15.00 10.12  9.96
98  98_HA_98_CA_98_CB_98_HB   15.30  6.63  9.90
105 105_HA_105_CA_105_CB_105_1HB 13.00  9.49  8.11
105 105_HA_105_CA_105_CB_105_2HB  2.60  4.34  5.60
106 106_HA_106_CA_106_CB_106_1HB 13.20  3.19  6.81
106 106_HA_106_CA_106_CB_106_2HB  3.10 10.43  7.01
111 111_HA_111_CA_111_CB_111_1HB  1.70  3.44  3.98
114 114_HA_114_CA_114_CB_114_2HB  1.80  3.65  6.09
116 116_HA_116_CA_116_CB_116_1HB  3.40  3.52  3.64
116 116_HA_116_CA_116_CB_116_2HB  9.80 10.34 10.33
118 118_HA_118_CA_118_CB_118_HB  4.90  2.44  2.67
119 119_HA_119_CA_119_CB_119_1HB  4.50  3.56  3.46
119 119_HA_119_CA_119_CB_119_2HB 13.30 10.77 10.78
120 120_HA_120_CA_120_CB_120_HB  4.50  2.62  2.69
123 123_HA_123_CA_123_CB_123_1HB  8.60 10.69 10.60
123 123_HA_123_CA_123_CB_123_2HB  0.30  3.71  3.99
124 124_HA_124_CA_124_CB_124_HB  4.20  3.19  7.66
127 127_HA_127_CA_127_CB_127_2HB  2.60  2.78  2.85
#
#
# C'-CA-CB-HB couplings
# Column 1: Residue number
# Column 2: Four atoms that define the dihedral, each labelled by residue
number and atom name
# Column 3: Experimental scalar coupling
# Column 4: Calculated scalar coupling from ff99SB simulation
# Column 5: Calculated scalar coupling from ff99SB-ILDN simulation
#
2   2_C_2_CA_2_CB_2_HB             2.60  1.48  1.65
3   3_C_3_CA_3_CB_3_1HB            1.40  1.70  1.23
3   3_C_3_CA_3_CB_3_2HB            3.60  1.98  1.99
6   6_C_6_CA_6_CB_6_1HB            3.70  1.07  1.08
6   6_C_6_CA_6_CB_6_2HB            2.60  1.33  1.31
8   8_C_8_CA_8_CB_8_1HB            8.50  4.11  4.09
8   8_C_8_CA_8_CB_8_2HB            3.10  1.34  1.35
12  12_C_12_CA_12_CB_12_1HB        1.80  1.13  1.13
12  12_C_12_CA_12_CB_12_2HB        2.20  1.84  1.83
15  15_C_15_CA_15_CB_15_1HB        2.20  1.21  1.18
17  17_C_17_CA_17_CB_17_1HB        1.80  3.07  2.90
17  17_C_17_CA_17_CB_17_2HB        2.20  1.68  1.67
```

18	18_C_18_CA_18_CB_18_1HB	8.30	3.95	3.68
20	20_C_20_CA_20_CB_20_1HB	8.10	4.11	4.11
20	20_C_20_CA_20_CB_20_2HB	4.40	1.34	1.32
23	23_C_23_CA_23_CB_23_1HB	3.00	1.51	2.10
23	23_C_23_CA_23_CB_23_2HB	2.60	1.58	1.58
24	24_C_24_CA_24_CB_24_1HB	1.30	1.10	1.22
24	24_C_24_CA_24_CB_24_2HB	7.40	2.84	2.39
27	27_C_27_CA_27_CB_27_1HB	0.30	3.34	1.24
27	27_C_27_CA_27_CB_27_2HB	1.10	1.84	1.56
30	30_C_30_CA_30_CB_30_1HB	7.70	3.88	3.89
30	30_C_30_CA_30_CB_30_2HB	2.30	1.18	1.04
33	33_C_33_CA_33_CB_33_1HB	1.10	4.10	4.09
33	33_C_33_CA_33_CB_33_2HB	12.50	1.30	1.31
34	34_C_34_CA_34_CB_34_1HB	1.10	1.18	1.87
34	34_C_34_CA_34_CB_34_2HB	2.20	1.72	1.59
38	38_C_38_CA_38_CB_38_1HB	0.70	1.41	1.41
38	38_C_38_CA_38_CB_38_2HB	3.00	1.43	1.45
39	39_C_39_CA_39_CB_39_1HB	8.10	4.11	3.73
39	39_C_39_CA_39_CB_39_2HB	2.20	1.34	1.42
40	40_C_40_CA_40_CB_40_HB	0.40	1.09	1.07
41	41_C_41_CA_41_CB_41_1HB	1.90	1.70	1.65
43	43_C_43_CA_43_CB_43_HB	1.80	1.69	1.36
47	47_C_47_CA_47_CB_47_HB	1.70	1.22	1.20
48	48_C_48_CA_48_CB_48_1HB	0.70	2.13	1.50
48	48_C_48_CA_48_CB_48_2HB	9.60	3.12	4.05
51	51_C_51_CA_51_CB_51_HB	1.70	0.83	0.80
52	52_C_52_CA_52_CB_52_2HB	1.10	1.31	1.20
53	53_C_53_CA_53_CB_53_1HB	2.60	1.17	1.21
53	53_C_53_CA_53_CB_53_2HB	1.90	1.77	1.69
55	55_C_55_CA_55_CB_55_HB	7.00	3.90	3.88
59	59_C_59_CA_59_CB_59_1HB	8.50	4.12	4.10
64	64_C_64_CA_64_CB_64_1HB	2.10	1.05	1.03
64	64_C_64_CA_64_CB_64_2HB	8.00	3.91	3.91
65	65_C_65_CA_65_CB_65_1HB	8.10	4.07	4.03
65	65_C_65_CA_65_CB_65_2HB	1.00	1.29	1.29
66	66_C_66_CA_66_CB_66_1HB	1.40	1.48	1.49
66	66_C_66_CA_66_CB_66_2HB	10.90	3.93	4.05
74	74_C_74_CA_74_CB_74_1HB	6.60	4.12	4.10
74	74_C_74_CA_74_CB_74_2HB	0.10	1.37	1.41
80	80_C_80_CA_80_CB_80_1HB	2.80	1.09	1.09
80	80_C_80_CA_80_CB_80_2HB	0.90	1.29	1.29
84	84_C_84_CA_84_CB_84_1HB	2.60	2.55	1.89
84	84_C_84_CA_84_CB_84_2HB	1.50	1.70	1.61
89	89_C_89_CA_89_CB_89_HB	3.30	0.90	0.82
92	92_C_92_CA_92_CB_92_HB	1.30	1.14	1.11
94	94_C_94_CA_94_CB_94_1HB	9.80	3.90	3.83
94	94_C_94_CA_94_CB_94_2HB	3.10	1.10	1.13
98	98_C_98_CA_98_CB_98_HB	1.80	1.11	1.00
105	105_C_105_CA_105_CB_105_1HB	1.10	1.64	2.14
105	105_C_105_CA_105_CB_105_2HB	1.40	1.63	1.62
106	106_C_106_CA_106_CB_106_1HB	3.00	4.00	2.68
106	106_C_106_CA_106_CB_106_2HB	3.00	1.49	1.55
116	116_C_116_CA_116_CB_116_1HB	8.50	3.94	3.94
116	116_C_116_CA_116_CB_116_2HB	2.20	1.46	1.46
118	118_C_118_CA_118_CB_118_HB	1.80	1.18	1.09
120	120_C_120_CA_120_CB_120_HB	3.00	1.44	1.41
123	123_C_123_CA_123_CB_123_1HB	1.10	1.36	1.44

123	123_C_123_CA_123_CB_123_2HB	1.80	1.46	1.38
124	124_C_124_CA_124_CB_124_HB	0.80	1.38	1.26
127	127_C_127_CA_127_CB_127_2HB	0.80	1.58	1.75

#
Rotamer distributions in the PDB and MD simulations.
#
m, p, and t refer to the 'minus', 'plus' and 'trans' rotamers
as defined by Lovell et al.
The values listed under PDB are the rotamets for residues in
helices as taken from Lovell et al
The values listed under ff99SB and ff99SB-ILDN, respectively,
are the rotamer distributions observed in simulations of
poly-alanine based helices as described in the manuscript
#

	PDB			ff99SB			ff99SB-ILDN		
	m	p	t	m	p	t	m	p	t
ARG	0.54	0.04	0.43	0.56	0.01	0.43	-	-	-
ASN	0.80	0.01	0.19	0.04	0.00	0.96	0.27	0.00	0.73
ASP	0.79	0.02	0.19	0.00	0.00	1.00	0.09	0.00	0.91
CYS	0.75	0.05	0.20	0.73	0.04	0.23	-	-	-
GLN	0.61	0.04	0.35	0.57	0.01	0.42	-	-	-
GLU	0.61	0.01	0.38	0.24	0.02	0.74	-	-	-
HIS	0.52	0.00	0.48	0.14	0.00	0.86	-	-	-
ILE	0.92	0.05	0.03	0.33	0.44	0.23	0.85	0.12	0.03
LEU	0.66	0.01	0.33	0.13	0.00	0.87	0.61	0.00	0.39
LYS	0.54	0.00	0.46	0.46	0.01	0.52	-	-	-
MET	0.72	0.02	0.26	0.42	0.02	0.56	-	-	-
PHE	0.41	0.01	0.58	0.18	0.00	0.82	-	-	-
SER	0.44	0.33	0.22	0.79	0.16	0.06	-	-	-
THR	0.75	0.25	0.00	0.75	0.25	0.00	-	-	-
TRP	0.39	0.03	0.58	0.29	0.02	0.70	-	-	-
TYR	0.42	0.01	0.57	0.15	0.00	0.85	-	-	-
VAL	0.07	0.02	0.91	0.30	0.07	0.63	-	-	-
